# Dimension-Specific Childhood Maltreatment and Cross-Sectional Markers of Illness Burden in Treatment-Resistant Depression: A Retrospective Single-Center Study

**DOI:** 10.3390/jcm15208182

**Published:** 2026-10-21

**Authors:** Giacomo d’Andrea, Luca Persico, Arianna Rosati, Clara Cavallotto, Emilia Esposito, Alessia Santeusanio, Michela D’Angola, Ilenia Rosa, Lorenzo Pio Padula, Antonio Arrè, Corinna Bozzelli, Alessandra De Franco, Lorenzo Varrica, Gianfranco Tamagnini, Mauro Pettorruso, Giovanni Martinotti

**Affiliations:** 1 Department of Neurosciences, Imaging and Clinical Sciences, Università degli Studi G. d’Annunzio, Chieti-Pescara, 66100 Chieti, Italy; giacomo.dandrea1993@gmail.com (G.d.); ariannarosati1996@gmail.com (A.R.); clara.cavallotto@laconchiglia.srl (C.C.); alessia.santeusanio@gmail.com (A.S.); micheladangola@gmail.com (M.D.); ilenia.rosa@unich.it (I.R.); lp.lorenzopadula@gmail.com (L.P.P.); antonio.arre3@gmail.com (A.A.); corinna.bozzelli99@gmail.com (C.B.); sandradef1305@gmail.com (A.D.F.); lorenzo.varrica@gmail.com (L.V.); mauro.pettorruso@hotmail.it (M.P.); giovanni.martinotti@gmail.com (G.M.); 2 Department of Mental Health, ASL 02 Lanciano-Vasto-Chieti, 66100 Chieti, Italy; gianfranco.tamagnini@asl2abruzzo.it; 3 Department of Pediatrics, Università degli Studi G. d’Annunzio, Chieti-Pescara, 66100 Chieti, Italy; emyesposito22@gmail.com; 4 Department of Pharmacy, Pharmacology and Clinical Science, University of Hertfordshire, Hatfield AL10 9AB, UK

**Keywords:** treatment-resistant depression, childhood maltreatment, Childhood Trauma Questionnaire, physical abuse, sexual abuse, emotional neglect, adverse childhood experiences, antidepressant treatment failure, precision psychiatry

## Abstract

**Background/Objectives**: We examined global and dimension-specific childhood maltreatment in relation to cross-sectional illness burden in treatment-resistant depression (TRD). **Methods**: This retrospective, single-center, case-only study included 104 records from November 2025 to June 2026. The Childhood Trauma Questionnaire–Short Form (CTQ-SF) was completed during TRD assessment. Primary outcomes were elapsed ongoing major depressive episode (MDE) duration and retrospectively reconstructed lifetime failed antidepressant-trial count; illness duration and previous MDEs were secondary. Models adjusted for age, sex, and extraction block; false discovery rate (FDR) correction covered 28 tests. **Results**: CTQ total and trauma load showed no significant linear associations. Per 5 points, physical abuse was associated with longer elapsed MDE duration (+50.3%; 95% CI, 24.0–82.2%; *p* < 0.001; q < 0.001) and more failed trials (IRR = 1.20; 95% CI, 1.10–1.30; *p* < 0.001; q < 0.001). Sexual abuse was associated with longer elapsed duration (+59.8%; 95% CI, 26.5–101.9%; *p* < 0.001; q = 0.001). Physical-abuse associations did not survive FDR correction in mutually adjusted subscale models; the failed-trial association also weakened after episode-duration adjustment. Emotional-neglect associations were inverse and require caution. **Conclusions**: These cross-sectional findings are hypothesis-generating and require prospective replication with standardized treatment histories and remission outcomes.

## 1. Introduction

Treatment-resistant depression (TRD) is generally defined by inadequate response to at least two antidepressant treatments delivered at adequate dose and duration, although operational definitions remain heterogeneous across research and clinical settings [1]. The construct identifies patients with substantial illness burden and a progressively lower probability of remission after successive treatment failures [2,3]. At the same time, TRD is not a unitary phenotype. Patients meeting the same resistance threshold may differ markedly in age at onset, recurrence, episode persistence, suicidality, comorbidity, symptom architecture, and psychosocial context [3,4]. Identifying factors that help explain this heterogeneity is therefore relevant not only to prognosis, but also to efforts to characterize treatment-resistant depressive illness more precisely.

Childhood maltreatment is among the most consistently replicated environmental determinants of depressive psychopathology. Meta-analytic evidence indicates that exposure to abuse or neglect is associated with a more adverse course of depression, including greater recurrence and persistence and poorer treatment outcomes [5]. A subsequent meta-analysis of 184 studies similarly described childhood maltreatment as a marker of an adult depressive phenotype characterized by earlier onset, greater severity, chronicity, and recurrence [6]. When analyses are restricted to studies using the Childhood Trauma Questionnaire (CTQ), the association with depression remains robust, but the magnitude of association varies across maltreatment dimensions, suggesting that abuse and neglect should not automatically be treated as interchangeable indicators of a single underlying exposure [7]. Longitudinal cohort data also support an association between childhood adversity and a more persistent course of depressive and anxiety disorders [8].

The relationship between childhood maltreatment and treatment response is more complex. The earlier meta-analysis by Nanni et al. found a higher likelihood of non-response or non-remission among depressed patients with a maltreatment history [5]. In the large international iSPOT-D effectiveness trial, childhood abuse—particularly physical, emotional, and sexual abuse occurring early in development—predicted poorer acute antidepressant outcomes across escitalopram, sertraline, and venlafaxine [9]. Earlier work in chronic depression had also suggested that childhood trauma may moderate relative response to psychotherapy and pharmacotherapy [10]. However, a more recent meta-analysis including up to 6830 participants found that, despite greater baseline severity, patients with childhood trauma improved substantially with evidence-based pharmacological and psychotherapeutic treatments and did not show a consistently smaller treatment effect than patients without trauma [11]. Consistent with that caution, secondary analyses of the CO-MED trial found no differential symptomatic or functional antidepressant response according to a history of childhood maltreatment, although side-effect burden was greater among exposed patients [12]. Conversely, a recent prospective cohort of 572 patients treated with selective serotonin reuptake inhibitors found lower response and remission among individuals reporting physical, emotional, or sexual abuse, whereas physical and emotional neglect were not associated with treatment outcome [13]. Thus, developmental adversity appears clinically important, but its relationship with treatment response is heterogeneous across cohorts and trauma dimensions and should not be reduced to a deterministic prediction of antidepressant failure.

Evidence focused specifically on TRD is still comparatively limited. In the French FACE-DR cohort, childhood maltreatment was associated with greater baseline depressive severity in patients with TRD, with particularly relevant signals for physical and sexual abuse; an apparent association with one-year remission was attenuated after adjustment for baseline severity [14]. A subsequent geriatric FACE-DR analysis again implicated physical abuse in greater depressive severity [15]. More recently, a population-based Swedish twin study found a graded association between adverse childhood experiences (ACEs) and the risk of TRD that remained evident in co-twin analyses, with physical neglect and sexual abuse showing among the largest type-specific associations [16]. In contrast, the international PROMPT study found that CTQ total score did not independently predict drug-resistant status, although several individual trauma dimensions were associated with depressive severity and showed heterogeneous relationships with resistance [17]. A recent systematic review similarly concluded that evidence directly linking childhood adversity to TRD remains limited and methodologically heterogeneous [18].

One potential explanation for these apparently discordant findings is the way childhood adversity is summarized. Cumulative indices are clinically intuitive and capture overall developmental burden, but they implicitly assume that heterogeneous experiences contribute in a broadly comparable direction. Dimensional frameworks instead distinguish experiences involving threat to physical integrity, such as physical or sexual abuse, from deprivation-related experiences characterized by the absence of expected emotional, social, or cognitive inputs [19]. Related work has proposed that maltreatment may generate clinically and neurobiologically meaningful “ecophenotypic” variation within psychiatric disorders [20]. Neurodevelopmental studies further indicate that the type and timing of maltreatment can be associated with partly distinct alterations in frontolimbic, salience, sensory, and reward-related systems [21,22]. These models do not establish trauma-specific depressive subtypes, but they provide a rationale for testing maltreatment dimensions separately rather than assuming that a global CTQ score contains all clinically relevant information.

This distinction is especially important once the analysis is restricted to patients who are already treatment resistant. Population-based studies ask whether childhood adversity increases the probability of developing TRD, whereas a case-only TRD study asks whether developmental exposures are associated with cross-sectional variation after the resistance threshold has already been crossed. Conditioning on a prior disease state may introduce index-event bias: factors contributing to entry into the selected cohort can become attenuated, inversely associated, or apparently null within that cohort [23,24]. Directionally unexpected coefficients must therefore be interpreted as potentially selection-induced rather than causal.

Accordingly, the present study examined both global and dimension-specific childhood-maltreatment measures in relation to four cross-sectional markers of illness burden in a real-world TRD cohort: elapsed duration of the ongoing major depressive episode (MDE) at assessment, overall illness duration, number of previous MDEs, and retrospectively reconstructed lifetime number of failed antidepressant trials. The objective was exploratory: to determine whether dimensions showed distinct association patterns, not to establish that dimension-specific models were superior to the CTQ total in prediction, explanation, or clinical utility. Because multiple exposures and outcomes were examined, all primary analyses were corrected for multiple testing and accompanied by complementary sensitivity analyses.

## 2. Materials and Methods

### 2.1. Study Design and Participants

This observational, retrospective, single-center, case-only study considered all eligible clinical records identified between November 2025 and June 2026 at the tertiary-level TRD Clinic of the ‘G. d’Annunzio’ University of Chieti–Pescara, SS. Annunziata Hospital, Chieti, Italy. The source database contained 134 records. Eligibility required adulthood, a current major depressive episode within a DSM-5-TR depressive-disorder diagnosis, and clinician-confirmed TRD. Diagnostic assessment was performed by psychiatrists experienced in TRD using a structured DSM-5-TR-based clinical interview; patients who did not meet TRD criteria were not included. The retrospective extract did not retain sufficiently standardized disorder-specific fields to permit a separate re-adjudication of occult bipolar-spectrum or mixed features, which is acknowledged as a limitation. After CTQ-SF quality screening, 104 participants formed the primary analytic cohort. Outcome-specific samples were *n* = 104 for elapsed ongoing-MDE duration and lifetime failed antidepressant trials, *n* = 99 for illness duration, and *n* = 93 for previous MDEs. The study is a case-only analysis of heterogeneity within TRD, not a case–control analysis of TRD risk. Calendar dates of database extraction and assembly of the internal blocks were not retained in the supplied extract and could not be independently reconstructed; the reported period refers to clinical cohort identification and assessment.

The internal extraction-block covariate distinguishes a principal block (*n* = 93) from a secondary export (*n* = 11) assembled retrospectively at the same center. Clinical assessments came from the same service; the secondary export omitted Item 28, contained no previous-MDE entries, and lacked age-at-first-MDE entries in five of the 11 records. No exact duplicate analytic records were detected. Because unique participant identifiers were not consistently retained in the secondary export, independent participant-level linkage could not be fully re-audited. At the source-record level, the principal block contained 120 records (93 included and 27 excluded for out-of-range CTQ values), whereas the secondary export contained 14 records (11 included and three excluded for missing clinical CTQ items). This identifies the block containing the affected records but does not establish the specific import event responsible for the coding errors.

TRD status was established during the specialist evaluation by psychiatrists experienced in TRD, integrating the structured diagnostic assessment, clinical interview, available records, major guideline definitions, and the Maudsley Staging Method [1,25,26,27]. A qualifying antidepressant trial required a guideline-recommended therapeutic dose, an adequate treatment duration of 8–12 weeks in the clinic’s assessment, and clinically adequate adherence. Adherence was assessed from the psychiatrist’s interview, longitudinal clinical documentation, and the treatment-history elements incorporated in Maudsley staging; pharmacy-dispensation or serum-level data were not uniformly available. TRD required non-response to at least two such trials.

The lifetime number of failed antidepressant trials was retrospectively reconstructed at the TRD evaluation and analyzed as a continuous marker of accumulated antidepressant exposure and non-response. It counted antidepressant medication trials across the lifetime course, not only the ongoing episode. Augmentation strategies, psychotherapy, electroconvulsive therapy, ketamine/esketamine, and other neuromodulation were considered in the overall clinical formulation and staging when documented but were not counted as failed antidepressant trials in this numeric outcome. Because this historical count and clinician-confirmed TRD drew on partly different record elements, a sensitivity analysis excluded the single participant with fewer than two recorded failed trials.

The study was conducted in accordance with the Declaration of Helsinki. The Ethics Committee reviewed the study protocol and all related documentation to ensure the protection of participants’ rights, privacy, and confidentiality. The study was approved by the local Territorial Ethics Committee of the Abruzzo Region (C.Et.R.A.) on 6 November 2025 (protocol no. 418308/25).

### 2.2. Clinical Variables and Outcomes

Sociodemographic and clinical variables included age, sex, marital status, age at first MDE, elapsed duration of the ongoing MDE, number of previous MDEs, psychiatric comorbidity, and lifetime failed antidepressant trials. At the specialist assessment, an experienced psychiatrist reconstructed these variables from the patient interview and available longitudinal records. Ambiguities were resolved clinically by the assessing psychiatrist; no independent blinded re-adjudication or formal inter-rater reliability study was performed. Illness duration was derived as current age minus age at first MDE. Current symptom-severity scores and treatment modalities were not recorded uniformly enough for harmonized analysis. Only an aggregate comorbidity indicator was consistently interpretable; PTSD, personality-disorder, and substance-use subtypes could not be reliably separated in the extract. The aggregate indicator was derived from the recorded numeric comorbidity field (0 = no recorded comorbidity; nonzero code = recorded comorbidity; missing retained as missing). A check against the raw numeric entries confirmed the reported counts, but did not provide independent diagnostic validation. Non-CTQ fields in the excluded records, including comorbidity and failed-trial counts, were not independently verified against original clinical records. Their tabulated values should therefore be interpreted as recorded database entries rather than independently validated clinical characteristics.

Four clinical outcomes were analyzed: (1) elapsed duration of the ongoing MDE at assessment, in months; (2) illness duration, in years; (3) number of previous MDEs; and (4) retrospectively reconstructed lifetime number of failed antidepressant trials. Elapsed ongoing-MDE duration is a prevalent cross-sectional measure and not a prospectively observed time-to-remission outcome. The four outcomes capture related but non-equivalent aspects of illness burden: current-episode persistence, time since first onset, recurrence, and accumulated antidepressant exposure/non-response.

### 2.3. Childhood Trauma Questionnaire

Childhood maltreatment was assessed at the same specialist visit as the TRD evaluation using the validated Italian-language 28-item Childhood Trauma Questionnaire–Short Form (CTQ-SF) [28,29,30]. The five clinical dimensions are emotional abuse, physical abuse, sexual abuse, emotional neglect, and physical neglect; each ranges from 5 to 25. The three-item Minimization/Denial (M/D) validity scale was scored separately and was positive when at least one M/D item received the maximum raw response of 5. CTQ total was the sum of the five clinical dimensions (range, 25–125); M/D items were not included.

**Figure 1:**
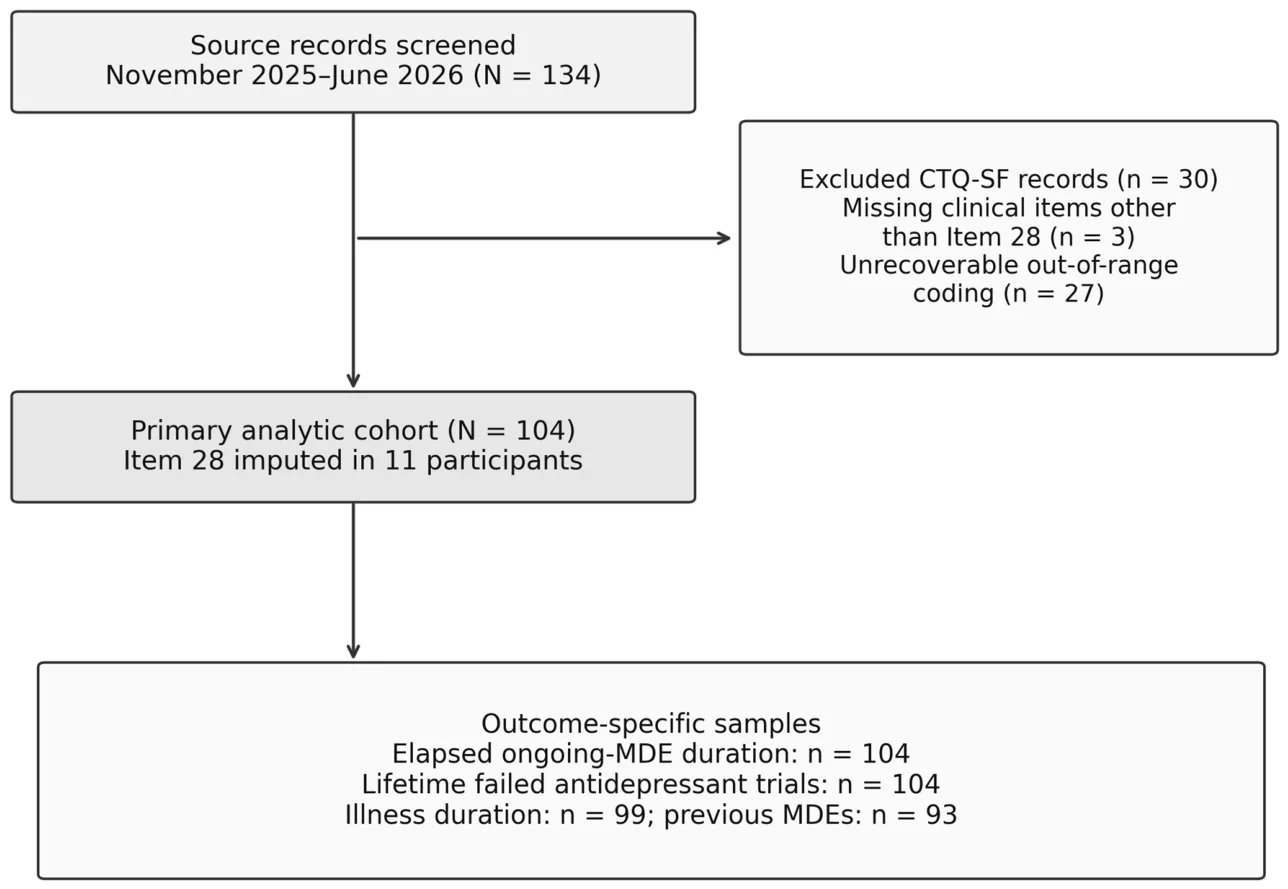
Participant flow from source records to outcome-specific analytic samples. The 27 out of range CTQ-SF records reflected unrecoverable import/coding errors. Item 28 was absent because of a systematic omission in the secondary extraction block; 11 included participants required primary-analysis replacement. CTQ-SF, Childhood Trauma Questionnaire–Short Form; MDE, major depressive episode.

Before scoring, CTQ responses were screened for completeness and the admissible range of 1–5. Thirty records were excluded: three lacked clinical CTQ-SF items other than Item 28 and 27 contained out-of-range values attributable to unrecoverable import/coding errors, not interpretable patient responses. Item 28 was absent from one internal extraction block because of a systematic import omission; among the 104 included participants, 11 were affected. Item 28 contributes to emotional neglect and is reverse scored. The primary analysis used the single-value replacement of 4 used in the original analysis. To assess imputation uncertainty, we added complete-case analyses and predictive-mean-matching multiple imputation with 50 datasets (five nearest donors, stochastic regression-parameter draws, and Rubin pooling; Table S7A). The imputation model included the other clinical CTQ items, age, sex, log ongoing-MDE duration, and failed-trial count. Because Item 28 was unobserved throughout the secondary block, this sensitivity assumes no residual block effect conditional on the measured predictors; it cannot verify missing at random. Standard reverse scoring was applied to Items 2, 5, 7, 13, 19, 26, and 28. Participant flow is shown in Figure 1.

In addition to CTQ total, trauma load was defined as the number of dimensions meeting standard moderate-to-severe thresholds: emotional abuse ≥ 13, physical abuse ≥ 10, sexual abuse ≥ 8, emotional neglect ≥ 15, and physical neglect ≥ 10 (range, 0–5) [28]. This threshold count was treated as a complementary descriptive exposure rather than a validated staging instrument.

### 2.4. Statistical Analysis

Analyses were performed in Python 3.12.14 using pandas 2.2.3, NumPy 2.3.5, and SciPy 1.17.0. Continuous variables are summarized by mean ± SD and median (IQR); categorical variables by n (%). Seven exposures were tested: CTQ total, trauma load, and the five CTQ subscales. Primary effects were scaled per 10 CTQ-total points, per additional trauma-load dimension, and per 5 subscale points; complementary per-SD estimates are provided in Table S2.

Each exposure was entered separately with age, sex, and internal extraction block, selected to address demographic and extraction-related heterogeneity while limiting model size. For previous-MDE models, extraction block was inestimable because this outcome existed only in the principal block. For elapsed ongoing-MDE duration, *log*(*Y*) = *β*_0_ + *β*_1_(*exposure*) + *β*_2_(*age*) + *β*_3_(*sex*) + *β*_4_(*block*) + *ε*. HC3 errors addressed heteroskedasticity and leverage in the modest sample; residual non-normality additionally motivated bootstrap sensitivity analyses. Exponentiated *β*_1_ was expressed as 100 × [*exp*(*β*_1_) − 1], the percent difference in conditional geometric mean duration. Illness duration used the analogous identity-link linear model with HC3 errors. Count outcomes used *log*[*E*(*Y*|*X*)] = *β*_0_ + *β*_1_(*exposure*) + *Σβ_k_X_k_* with leverage-adjusted sandwich errors; *exp*(*β*_1_) is an incidence-rate ratio. Negative-binomial models addressed overdispersion. Ordinal failed-trial sensitivity excluded the single value of 1 and pooled counts ≥ 6 to avoid complete separation at the highest threshold in the secondary block. Proportional odds were assessed by exposure-specific and omnibus Wald tests using the joint, participant-level cross-threshold covariance. The ordinal model was not treated as confirmatory if the omnibus assumption failed. The Poisson models were working log-mean regressions within the selected TRD cohort, not a model of the selection-induced lower truncation of failed-trial counts. Linear-model inference used residual-df *t* tests; count-model inference used normal-reference Wald tests.

The primary family comprised 28 exposure–outcome tests and used Benjamini–Hochberg FDR correction. Diagnostics included count distributions, Pearson dispersion, log-model residual tests, CTQ-subscale correlations, and variance-inflation factors. Linearity was explored with three-knot restricted cubic splines at the 10th, 50th, and 90th percentiles when distinct knots were estimable; because physical- and sexual-abuse medians were at the floor, their threshold analyses were more informative. Sensitivities comprised M/D adjustment and exclusion, complete-case and 50-dataset multiple-imputation analyses for Item 28, principal-block restriction, illness-duration and ongoing-MDE-duration adjustment where relevant, ≥2 failed-trial restriction, mutually adjusted subscales, clinical cutoffs, per-SD scaling, negative-binomial and ordinal models, 2000 bootstrap resamples, leave-one-out analyses, and CTQ-by-sex interactions. Baseline severity, socioeconomic circumstances, treatment access, medication-exposure details, and current adversity were unavailable or insufficiently harmonized and could not be covaried. All tests were two-sided; exact *p* values are reported to three decimals, with *p* < 0.001 for smaller values. All analyses were exploratory; sensitivity analyses comprised those in the original submission and additional reviewer-requested checks. An aggregate psychiatric-comorbidity-adjusted family was also reported. Included-versus-excluded and outcome-missingness comparisons used Mann–Whitney tests for continuous variables and χ^2^ or Fisher exact tests for categorical variables; these descriptive *p* values were unadjusted. Secondary q values were corrected only within the family identified in each table. During the first revision, inferential results were regenerated from the same corrected source dataset using the explicitly specified robust covariance calculations and reference distributions: HC3 covariance with residual-degrees-of-freedom *t* tests for linear models and leverage-adjusted sandwich covariance with normal-reference Wald tests for count models. This revision of the inferential workflow changed some confidence intervals, *p* values, and FDR-adjusted q values without necessarily changing rounded point estimates.

## 3. Results

### 3.1. Sample Characteristics

Of 134 source records, 30 were excluded after CTQ-SF quality screening (Figure 1), leaving 104 participants. Excluded records had fewer recorded failed antidepressant trials than included records (mean 2.33 vs. 4.09; *p* < 0.001) and more previous MDEs among those with data (3.52 vs. 2.63; *p* = 0.016), suggesting possible selection or data-quality differences (Table S4A). Recorded psychiatric comorbidity was also more frequent among excluded records (29/30, 96.7%) than among included participants with data (40/103, 38.8%; *p* < 0.001). In the analytic sample, mean age was 55.0 ± 13.4 years, 44 participants (42.3%) were female, and 40/103 (38.8%) had at least one recorded psychiatric comorbidity. Mean age at first MDE was 36.8 ± 14.7 years. Mean CTQ total was 47.4 ± 14.6 and mean trauma load was 1.62 ± 1.33 dimensions. Elapsed ongoing-MDE duration was 24.0 ± 17.5 months; illness duration, 18.3 ± 12.1 years (*n* = 99); previous MDEs, 2.63 ± 2.15 (*n* = 93); and lifetime failed trials, 4.09 ± 2.22 (range, 1–12) (Table 1). Participants missing secondary outcomes had higher CTQ scores than those with observed outcomes (Table S4B), further limiting interpretation of those secondary analyses. These descriptive contrasts require caution because non-CTQ fields in the excluded records were not independently verified; they may reflect clinical differences, additional coding problems, or both.

### 3.2. Global Childhood-Trauma Burden

No statistically significant linear association was detected between CTQ total or trauma load and any of the four outcomes after global FDR correction (all q = 0.979; Table 2 and Table S1). For example, per 10 CTQ-total points, the adjusted difference in elapsed ongoing-MDE duration was +0.5% (95% CI, −17.7% to +22.8%; *p* = 0.962), an interval that does not exclude modest effects. Exploratory spline analyses suggested nonlinearity for CTQ total and trauma load in relation to elapsed ongoing-MDE duration; these post hoc findings are reported in Table S12 and require independent replication.

### 3.3. Dimension-Specific Associations with Ongoing-MDE Duration and Failed Antidepressant Trials

**Figure 2:**
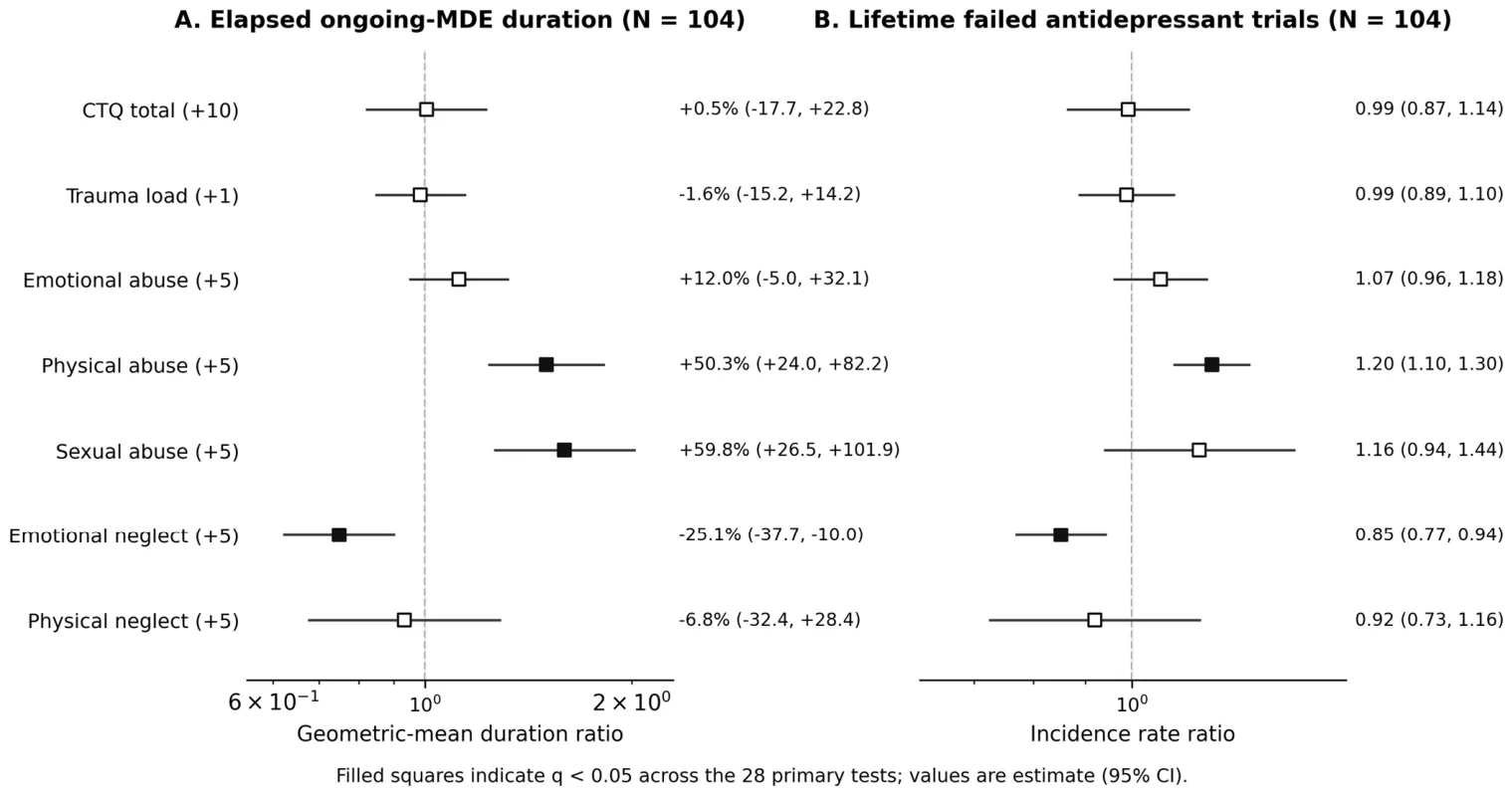
Adjusted associations with elapsed ongoing-MDE duration and lifetime failed antidepressant trials (*n* = 104 for both panels). Values printed beside each marker are adjusted estimates (95% CIs). CTQ total is scaled per 10 points, trauma load per dimension, and subscales per 5 points. Filled markers denote q < 0.05 across the 28 primary tests. Models adjust for age, sex, and internal extraction block. CTQ, Childhood Trauma Questionnaire; IRR, incidence-rate ratio; MDE, major depressive episode.

Dimension-specific models showed heterogeneous cross-sectional associations (Table 2; Figure 2). Per 5 physical-abuse points, elapsed ongoing-MDE duration was 50.3% longer (95% CI, 24.0–82.2%; *p* < 0.001; q < 0.001). Per 5 sexual-abuse points, it was 59.8% longer (95% CI, 26.5–101.9%; *p* < 0.001; q = 0.001). Emotional neglect showed an inverse association (−25.1%; 95% CI, −37.7% to −10.0%; *p* = 0.002; q = 0.013), which was not interpreted as protective.

Physical abuse was also associated with the retrospectively recorded lifetime failed-trial count (IRR = 1.20 per 5 points; 95% CI, 1.10–1.30; *p* < 0.001; q < 0.001), and emotional neglect showed an inverse association (IRR = 0.85; 95% CI, 0.77–0.94; *p* = 0.002; q = 0.012). Sexual abuse was not significantly associated with failed trials in the corrected analysis (IRR = 1.16; 95% CI, 0.94–1.44; *p* = 0.163; q = 0.486). No other dimension-specific failed-trial association survived correction.

### 3.4. Other Clinical-Course Outcomes and Sensitivity Analyses

No exposure survived global FDR correction for illness duration or previous MDE count (Table S1). Physical neglect was nominally associated with fewer previous MDEs (IRR = 0.78; 95% CI, 0.62–0.97; *p* = 0.029; q = 0.133). In the mutually adjusted subscale model, physical neglect was associated with shorter illness duration (−6.05 years per 5 points; 95% CI, −9.91 to −2.18; *p* = 0.003; q = 0.022 across the 20 exploratory tests in Table S9), whereas its single-exposure estimate was nonsignificant (−2.94 years; 95% CI, −6.12 to +0.25; *p* = 0.070). Given subscale correlations (maximum Spearman ρ = 0.64) and VIF up to 3.08, this conditional coefficient was treated as exploratory.

Sensitivity analyses generally preserved the direction of the physical-abuse associations while quantifying their uncertainty (Tables S2–S18). The failed-trial outcome showed limited Poisson overdispersion; negative-binomial estimates remained compatible (IRR = 1.20; 95% CI, 1.06–1.35; *p* = 0.004). The ordinal model (*n* = 103; counts ≥6 pooled) yielded a physical-abuse common OR of 2.94 (95% CI, 1.53–5.63; *p* = 0.001). However, the omnibus proportional-odds test rejected the common-slope assumption (*p* < 0.001), despite no detected heterogeneity of the physical-abuse slope alone (*p* = 0.307). The ordinal estimate is therefore exploratory and is not used as confirmatory evidence. Elapsed ongoing-MDE duration and failed trials were modestly correlated (Spearman ρ = 0.254; *p* = 0.009). After adjustment for log ongoing-MDE duration, the physical-abuse/failed-trial association attenuated but remained nominally evident (IRR = 1.15; 95% CI, 1.03–1.28; *p* = 0.013; q = 0.055 within the seven-test exploratory family in Table S17). This association remained nominally significant but did not survive within-family FDR correction, limiting evidence that it represents a distinct signal.

In 2000 bootstrap samples, the 95% intervals were +22.4% to +80.9% for physical abuse/elapsed duration and 1.08–1.31 for physical abuse/failed trials. Leave-one-out physical-abuse duration estimates ranged from +47.4% to +61.6% (*p* range < 0.001–0.002; 0/104 iterations lost nominal significance; maximum absolute change, 11.3 percentage points). Sexual-abuse duration estimates ranged from +53.6% to +66.6% (*p* < 0.001–0.060; 1/104 lost significance), demonstrating greater case sensitivity. Complete-case and multiple-imputation analyses for Item 28 were materially consistent with primary estimates. No CTQ-by-sex interaction survived FDR correction. Full, rather than selected, sensitivity results are reported in the Supplement.

## 4. Discussion

### 4.1. Principal Findings

This retrospective study examined whether cumulative and dimension-specific CTQ measures were associated with cross-sectional illness-burden markers among patients already meeting TRD criteria. The design does not compare predictive performance or establish incremental clinical value.

Three observations merit cautious consideration. First, no significant linear association was detected for CTQ total or trauma load, although confidence intervals allowed modest effects and exploratory spline results raised the possibility of nonlinearity. Second, physical-abuse scores were associated with longer elapsed ongoing-MDE duration and a higher retrospectively recorded lifetime failed-trial count; sexual abuse was associated with elapsed duration only. Third, emotional-neglect coefficients were inverse and should not be interpreted as protective. All findings are associative and vulnerable to selection, measurement, and residual confounding.

Overall, this single-cohort analysis suggests that maltreatment dimensions may show different association patterns, but it does not establish superiority of dimension-specific assessment, prognostic improvement, or clinical utility.

### 4.2. Physical and Sexual Abuse: Associations with Ongoing Episode Duration and Treatment Failure

The physical-abuse associations were the most consistent within the analyzed specifications. They are compatible with the literature linking maltreatment to persistent depression and poorer treatment outcomes [5,6,8,9,13,14,15,31]. Nevertheless, convergence across related sensitivity analyses cannot remove uncertainty from retrospective outcome reconstruction, selection into TRD, unmeasured current severity, sparse high exposures, or residual confounding. The present data therefore complement, rather than extend causally, earlier findings and should be considered hypothesis-generating. In the mutually adjusted five-subscale models, the physical-abuse coefficients retained their direction but did not survive the 20-test exploratory FDR correction (elapsed duration, +38.4%; *p* = 0.023; q = 0.117; failed trials, IRR = 1.19; *p* = 0.052; q = 0.208; Table S9). The single-exposure associations therefore do not establish an effect specific to physical abuse independent of correlated maltreatment dimensions.

Sexual abuse was associated with elapsed ongoing-MDE duration but not with the corrected lifetime failed-trial count. Because elapsed duration is a prevalent cross-sectional measure influenced by treatment timing, access, tolerability, and psychosocial maintenance, it cannot establish prospective prolongation of remission. The estimate also showed greater leave-one-out sensitivity and a wide bootstrap interval. Replication is therefore particularly important.

Physical and sexual abuse are commonly conceptualized as threat-related experiences within dimensional models of adversity [19]. Threat exposure has been associated with altered emotional learning, salience processing, stress responsivity, and frontolimbic development, while maltreatment more broadly has been linked to structural and functional alterations in prefrontal, cingulate, amygdalar, hippocampal, sensory, and reward-related systems [20,21,22,32]. An integrative review of meta-analytic evidence further identified a convergent pattern of blunted cortisol reactivity, low-grade inflammation, heightened amygdala responsivity to negative information, and reduced hippocampal volume following early-life adversity [33]. Human studies also support long-lasting alterations of hypothalamic–pituitary–adrenal-axis regulation following childhood trauma [34] and an association between childhood maltreatment and persistent inflammatory activation in adulthood [35,36]; a systematic review specifically in MDD likewise identified childhood adversity as a potential contributor to inflammatory heterogeneity [37]. These mechanisms are plausible pathways through which early threat could contribute to depressive persistence or reduced treatment responsiveness, but they remain hypotheses in the present context: no biological measure was available in this study, and none of these pathways is specific to TRD.

### 4.3. Why Was Global CTQ Burden Null?

The absence of statistically significant linear associations for CTQ total or trauma load should not be interpreted as proof of no relationship. For CTQ total and elapsed duration, the 95% CI ranged from −17.7% to +22.8% per 10 points. Population studies address TRD incidence, whereas this analysis conditions on an established resistant phenotype. Index-event bias can attenuate or reverse associations among causes of cohort entry [23,24]. The exploratory spline signals also suggest that a monotonic linear summary may be incomplete, although these post hoc findings require confirmation.

The total-score result is compatible with PROMPT, where CTQ total did not independently predict drug-resistant status [17], but the present study did not formally compare model superiority. Cumulative and dimension-specific measures may answer different questions; our data only show that their estimated association patterns differed in this cohort.

### 4.4. The Emotional-Neglect Association Requires Cautious Interpretation

The inverse association involving emotional neglect is the most counterintuitive result and should be regarded as hypothesis-generating. It is inconsistent with the broader literature, in which neglect is associated with depressive vulnerability, persistence, and—in some studies—poorer treatment outcome [6,7,18]. It therefore should not be described as protective. Several non-causal explanations are plausible. First, the CTQ is a retrospective self-report measure, and under-reporting or minimization can influence estimates of childhood maltreatment; Minimization/Denial is common enough to affect CTQ validity in large samples [38]. Consistent with this concern, the inverse association between emotional neglect and ongoing-MDE duration attenuated from −25.1% in the primary model to −14.0% and became nonsignificant when participants with evidence of Minimization/Denial were excluded. The failed-trial association was more persistent, but its unexpected direction still warrants restraint.

Second, conditioning on TRD may induce index-event bias: even independent risk factors can become inversely associated after selection on a shared disease event [23,24]. Third, CTQ dimensions co-occur, and conditional coefficients may be altered by shared variance. Fourth, occult bipolar-spectrum or mixed features are a clinically relevant source of apparent antidepressant resistance [39,40,41,42]. Although DSM-5-TR diagnostic assessment was conducted by TRD psychiatrists, the retrospective extract did not permit independent re-adjudication of these features. Together with residual confounding and uneven exposure distributions, these limitations preclude causal or protective interpretation of emotional neglect.

### 4.5. Clinical and Research Implications

The clinical implication is not that childhood maltreatment should be used as a stand-alone treatment-selection rule. The best available meta-analysis indicates that patients with childhood trauma still derive substantial benefit from evidence-based pharmacotherapy and psychotherapy [11]. Historical findings suggesting differential benefit of psychotherapy in chronically depressed patients with childhood trauma [10], antidepressant-moderation signals from iSPOT-D [9], the null differential response observed in CO-MED [12], and the more recent abuse-specific associations reported after SSRI treatment [13] collectively illustrate why the field is not yet ready for trauma-based treatment allocation. Trauma history should therefore inform formulation, prognosis, and the need for trauma-informed care without creating therapeutic pessimism.

At the research level, the findings justify further evaluation—not clinical adoption—of dimension-specific developmental phenotyping. No treatment-by-trauma interaction, prospective response endpoint, model calibration, decision curve, or incremental-prediction analysis was performed. Prospective studies should determine whether trauma dimensions add value beyond established clinical variables before they are used for prognostic stratification or treatment matching.

### 4.6. Strengths and Limitations

Several limitations frame interpretation. The retrospective, single-center, case-only design precludes causal inference. CTQ-SF was retrospective and susceptible to recall, response-style, and M/D effects. The ongoing MDE was still in progress at assessment, making elapsed duration vulnerable to length-biased sampling. Thirty of the 134 records were excluded for CTQ quality problems; recorded differences between excluded and included records suggest possible selection bias, but non-CTQ fields in excluded records were not independently verified, so additional coding errors may also contribute to these contrasts. The systematic Item 28 omission in one extraction block required a single-value replacement; complete-case and multiple-imputation analyses were reassuring but rest on different missingness assumptions. Illness-duration and previous-MDE outcomes were missing for 5 and 11 participants, respectively, entirely in the secondary extraction block. Historical treatment failures were reconstructed from interview and records and may be misclassified; a simple antidepressant count incompletely captures dose, adherence, sequencing, augmentation, psychotherapy, ECT, ketamine/esketamine, neuromodulation, and multidimensional resistance [26,43]. Current severity, socioeconomic circumstances, treatment access, current adversity, and harmonized treatment modality were unavailable. The retrospective extract did not support independent re-adjudication of occult bipolar-spectrum or mixed features. The modest sample, sparse high physical/sexual-abuse exposure, wide intervals, and limited power constrain categorical, interaction, spline, and mutually adjusted analyses. Finally, selection on TRD may induce index-event bias [23,24]. No exact duplicate records were found, but inconsistent retention of unique identifiers limited a complete independent participant-linkage audit. The ordinal sensitivity violated the omnibus proportional-odds assumption and was not used as confirmatory support.

Strengths include transparent separation of cumulative and subscale measures, four complementary outcomes, global FDR control, effect scaling, complete reporting of all primary models, and sensitivity analyses addressing imputation, distributions, influence, nonlinearity, and internal extraction blocks. These safeguards improve transparency but do not convert the observational associations into causal or clinically actionable evidence.

## 5. Conclusions

In this retrospective, single-center TRD cohort, physical- and sexual-abuse subscale scores were associated with longer elapsed duration of an ongoing depressive episode, while physical abuse was also associated with a higher retrospectively recorded lifetime count of failed antidepressant trials. The physical-abuse associations did not survive FDR correction in mutually adjusted subscale models, and the failed-trial association weakened after adjustment for ongoing-episode duration, also failing its exploratory FDR correction. These associative, hypothesis-generating findings do not establish causality, prognostic superiority, or treatment utility. Replication is required in prospective cohorts with standardized diagnostic and treatment-history assessment, repeated severity measurement, and longitudinal time-to-remission outcomes.

## Figures and Tables

**Table 1:** Sociodemographic, clinical, and childhood-maltreatment characteristics of the primary sample.

Characteristic	N	Value
Sociodemographic characteristics		
Age, years	104	55.0 ± 13.4; median 57.0 (IQR 48.0–64.3)
Female sex	104	44 (42.3%)
Marital status	104	Single 20 (19.2%); married 73 (70.2%); widowed 11 (10.6%)
Childhood maltreatment		
CTQ total score	104	47.4 ± 14.6; median 49 (IQR 36–57)
Trauma load, dimensions	104	1.62 ± 1.33; median 2 (IQR 0–3)
Emotional abuse	104	9.49 ± 4.95
Physical abuse	104	6.99 ± 3.52
Sexual abuse	104	6.15 ± 2.41
Emotional neglect	104	14.58 ± 5.65
Physical neglect	104	10.23 ± 4.36
Clinical course		
Age at first MDE, years	99	36.8 ± 14.7; median 35.0 (IQR 25.5–46.5)
Any recorded psychiatric comorbidity	103	40 (38.8%); 1 missing
Current MDE duration, months	104	24.0 ± 17.5; median 18 (IQR 12–36)
Illness duration, years	99	18.3 ± 12.1; median 18 (IQR 7–27.5)
Previous MDEs, n	93	2.63 ± 2.15; median 2 (IQR 1–3)
Lifetime failed antidepressant trials, n	104	4.09 ± 2.22; median 4 (IQR 2–5); range 1–12

Values are mean ± SD or n (%), with medians and IQRs additionally shown for selected continuous variables. CTQ, Childhood Trauma Questionnaire; IQR, interquartile range; MDE, major depressive episode; SD, standard deviation.

**Table 2:** Primary associations of global and dimension-specific childhood-trauma measures with ongoing-MDE duration and lifetime failed antidepressant trials.

CTQ Measure	Increase	Ongoing MDE: % Difference (95% CI)	p; q	Failed Trials: IRR (95% CI)	p; q
CTQ total	+10 points	+0.5% (−17.7% to +22.8%)	0.962; 0.979	0.99 (0.87–1.14)	0.897; 0.979
Trauma load	+1 dimension	−1.6% (−15.2% to +14.2%)	0.835; 0.979	0.99 (0.89–1.10)	0.819; 0.979
Emotional abuse	+5 points	+12.0% (−5.0% to +32.1%)	0.173; 0.486	1.07 (0.96–1.18)	0.229; 0.582
Physical abuse	+5 points	+50.3% (+24.0% to +82.2%)	<0.001; <0.001	1.20 (1.10–1.30)	<0.001; <0.001
Sexual abuse	+5 points	+59.8% (+26.5% to +101.9%)	<0.001; 0.001	1.16 (0.94–1.44)	0.163; 0.486
Emotional neglect	+5 points	−25.1% (−37.7% to −10.0%)	0.002; 0.013	0.85 (0.77–0.94)	0.002; 0.012
Physical neglect	+5 points	−6.8% (−32.4% to +28.4%)	0.664; 0.979	0.92 (0.73–1.16)	0.485; 0.979

Effects are scaled per the increment shown. All models adjusted for age, sex, and internal extraction block. Ongoing-MDE effects are exponentiated log-linear estimates expressed as percentage differences in conditional geometric mean elapsed duration; failed-trial effects are IRRs. q values are Benjamini–Hochberg FDR-adjusted across all 28 primary tests. Exact β coefficients and robust SEs for every primary model are reported in Table S1. CTQ, Childhood Trauma Questionnaire; FDR, false discovery rate; IRR, incidence-rate ratio; MDE, major depressive episode.

## Data Availability

The data presented in this study are available from the corresponding author upon reasonable request. The data are not publicly available because they contain sensitive clinical information and are subject to privacy and ethical restrictions.

## Supplementary Materials

The following supporting information can be downloaded at: https://www.mdpi.com/article/10.3390/jcm15208182/s1, Supplementary Tables S1A-S1B-S1C-S1D-S2-S3-S4A-S4B-S5A-S5B-S5C-S6A-S6B-S6C-S6D-S6E-S7A-S7B-S8-S9-S10-S11-S12-S13-S14-S15-S16-S17-S18 provide the complete primary family, per-SD and cutoff analyses, cohort-flow comparisons, diagnostics, imputation analyses, full sensitivity models, nonlinearity assessments, and influence analyses.

## References

[1] Sforzini L., Worrell C., Kose M., Anderson I.M., Aouizerate B., Arolt V., Bauer M., Baune B.T., Blier P., Cleare A.J. (2022). A Delphi-method-based consensus guideline for definition of treatment-resistant depression for clinical trials. Mol. Psychiatry.

[2] Rush A.J., Trivedi M.H., Wisniewski S.R., Nierenberg A.A., Stewart J.W., Warden D., Niederehe G., Thase M.E., Lavori P.W., Lebowitz B.D. (2006). Acute and longer-term outcomes in depressed outpatients requiring one or several treatment steps: A STAR*D report. Am. J. Psychiatry.

[3] O’Connor S.J., Hewitt N., Kuc J., Orsini L.S. (2024). Predictors and risk factors of treatment-resistant depression: A systematic review. J. Clin. Psychiatry.

[4] Maj M., Stein D.J., Parker G., Zimmerman M., Fava G.A., De Hert M., Demyttenaere K., McIntyre R.S., Widiger T., Wittchen H. (2020). The clinical characterization of the adult patient with depression aimed at personalization of management. World Psychiatry.

[5] Nanni V., Uher R., Danese A. (2012). Childhood maltreatment predicts unfavorable course of illness and treatment outcome in depression: A meta-analysis. Am. J. Psychiatry.

[6] Nelson J., Klumparendt A., Doebler P., Ehring T. (2017). Childhood maltreatment and characteristics of adult depression: Meta-analysis. Br. J. Psychiatry.

[7] Humphreys K.L., LeMoult J., Wear J.G., Piersiak H.A., Lee A., Gotlib I.H. (2020). Child maltreatment and depression: A meta-analysis of studies using the Childhood Trauma Questionnaire. Child Abus. Negl..

[8] Hovens J.G.F.M., Giltay E.J., Wiersma J.E., Spinhoven P., Penninx B.W.J.H., Zitman F.G. (2012). Impact of childhood life events and trauma on the course of depressive and anxiety disorders. Acta Psychiatr. Scand..

[9] Williams L.M., Debattista C., Duchemin A.-M., Schatzberg A.F., Nemeroff C.B. (2016). Childhood trauma predicts antidepressant response in adults with major depression: Data from the randomized international study to predict optimized treatment for depression. Transl. Psychiatry.

[10] Nemeroff C.B., Heim C.M., Thase M.E., Klein D.N., Rush A.J., Schatzberg A.F., Ninan P.T., McCullough J.P., Weiss P.M., Dunner D.L. (2003). Differential responses to psychotherapy versus pharmacotherapy in patients with chronic forms of major depression and childhood trauma. Proc. Natl. Acad. Sci. USA.

[11] Kuzminskaite E., Gathier A.W., Cuijpers P., Penninx B.W.J.H., Ammerman R.T., Brakemeier E.-L., Bruijniks S., Carletto S., Chakrabarty T., Douglas K. (2022). Childhood Trauma Meta-Analysis Study Group. Treatment efficacy and effectiveness in adults with major depressive disorder and childhood trauma history: A systematic review and meta-analysis. Lancet Psychiatry.

[12] Medeiros G.C., Prueitt W.L., Rush A.J., Minhajuddin A., Czysz A.H., Patel S.S., Trombello J., Trivedi M.H. (2021). Impact of childhood maltreatment on outcomes of antidepressant medication in chronic and/or recurrent depression. J. Affect. Disord..

[13] Li Y., Chen Y., Jiang Y., Wang W., Guo L., Fan B., Liu Y., Zhang H., Lin X., Teopiz K.M. (2025). Associations of childhood trauma with remission and treatment response after 12 weeks of selective serotonin reuptake inhibitor treatment in patients with major depressive disorder. Gen. Hosp. Psychiatry.

[14] Yrondi A., Aouizerate B., Bennabi D., Richieri R., D’AMato T., Bellivier F., Bougerol T., Horn M., Camus V., Courtet P. (2020). Childhood maltreatment and clinical severity of treatment-resistant depression in a French cohort of outpatients (FACE-DR): One-year follow-up. Depress. Anxiety.

[15] Yrondi A., Arbus C., Bennabi D., D’aMato T., Bellivier F., Bougerol T., Camus V., Courtet P., Doumy O., Genty J.-B. (2021). Relationship between childhood physical abuse and clinical severity of treatment-resistant depression in a geriatric population. PLoS ONE.

[16] Xiong Y., Lindersten P., Gong T., Magnusson P.K.E., Liu S., Lu Y. (2026). Adverse childhood experiences and treatment-resistant depression. JAMA Netw. Open.

[17] Ferensztajn-Rochowiak E., Rybakowski F., Michalak M., Kopeć D., Minelli A., Potier M.-C., Dierssen M., Manchia M., Bel J.P., Squassina A. (2026). PROMPT Study Group. The effect of childhood trauma on the severity of depressive symptoms and drug resistance in major depressive disorder—Results from the international PROMPT study. J. Affect. Disord..

[18] Dos Santos M.A.B., Guerra A.E.P., Sayão M.M.A., Selani A.L.S., Betarelli B.C., Siqueira I.F., Forgerini M. (2026). The role of adverse childhood experiences in the development of major depressive disorder and treatment-resistant depression. Child Psychiatry Hum. Dev..

[19] McLaughlin K.A., Sheridan M.A., Lambert H.K. (2014). Childhood adversity and neural development: Deprivation and threat as distinct dimensions of early experience. Neurosci. Biobehav. Rev..

[20] Teicher M.H., Samson J.A. (2013). Childhood maltreatment and psychopathology: A case for ecophenotypic variants as clinically and neurobiologically distinct subtypes. Am. J. Psychiatry.

[21] Teicher M.H., Samson J.A. (2016). Annual Research Review: Enduring neurobiological effects of childhood abuse and neglect. J. Child Psychol. Psychiatry.

[22] Teicher M.H., Samson J.A., Anderson C.M., Ohashi K. (2016). The effects of childhood maltreatment on brain structure, function and connectivity. Nat. Rev. Neurosci..

[23] Dahabreh I.J., Kent D.M. (2011). Index event bias as an explanation for the paradoxes of recurrence risk research. JAMA.

[24] Smits L.J.M., van Kuijk S.M.J., Leffers P., Peeters L.L., Prins M.H., Sep S.J.S. (2013). Index event bias—A numerical example. J. Clin. Epidemiol..

[25] McIntyre R.S., Alsuwaidan M., Baune B.T., Berk M., Demyttenaere K., Goldberg J.F., Gorwood P., Ho R., Kasper S., Kennedy S.H. (2023). Treatment-resistant depression: Definition, prevalence, detection, management, and investigational interventions. World Psychiatry.

[26] Fekadu A., Wooderson S., Donaldson C., Markopoulou K., Masterson B., Poon L., Cleare A.J. (2009). A multidimensional tool to quantify treatment resistance in depression: The Maudsley Staging Method. J. Clin. Psychiatry.

[27] Fekadu A., Donocik J.G., Cleare A.J. (2018). Standardisation framework for the Maudsley Staging Method for treatment resistance in depression. BMC Psychiatry.

[28] Bernstein D.P., Stein J.A., Newcomb M.D., Walker E., Pogge D., Ahluvalia T., Stokes J., Handelsman L., Medrano M., Desmond D. (2003). Development and validation of a brief screening version of the Childhood Trauma Questionnaire. Child Abus. Negl..

[29] Sacchi C., Vieno A., Simonelli A. (2018). Italian validation of the Childhood Trauma Questionnaire–Short Form on a college group. Psychol. Trauma.

[30] Innamorati M., Erbuto D., Venturini P., Fagioli F., Ricci F., Lester D., Amore M., Girardi P., Pompili M. (2016). Factorial validity of the Childhood Trauma Questionnaire in Italian psychiatric patients. Psychiatry Res..

[31] Miniati M., Rucci P., Benvenuti A., Frank E., Buttenfield J., Giorgi G., Cassano G.B. (2010). Clinical characteristics and treatment outcome of depression in patients with and without a history of emotional and physical abuse. J. Psychiatr. Res..

[32] McCrory E.J., Gerin M.I., Viding E. (2017). Annual Research Review: Childhood maltreatment, latent vulnerability and the shift to preventative psychiatry—The contribution of functional brain imaging. J. Child Psychol. Psychiatry.

[33] Hakamata Y., Suzuki Y., Kobashikawa H., Hori H. (2022). Neurobiology of early life adversity: A systematic review of meta-analyses towards an integrative account of its neurobiological trajectories to mental disorders. Front. Neuroendocrinol..

[34] Heim C., Newport D.J., Mletzko T., Miller A.H., Nemeroff C.B. (2008). The link between childhood trauma and depression: Insights from HPA axis studies in humans. Psychoneuroendocrinology.

[35] Danese A., Pariante C.M., Caspi A., Taylor A., Poulton R. (2007). Childhood maltreatment predicts adult inflammation in a life-course study. Proc. Natl. Acad. Sci. USA.

[36] Danese A., Moffitt T.E., Pariante C.M., Ambler A., Poulton R., Caspi A. (2008). Elevated inflammation levels in depressed adults with a history of childhood maltreatment. Arch. Gen. Psychiatry.

[37] Gill H., El-Halabi S., Majeed A., Gill B., Lui L.M.W., Mansur R.B., Lipsitz O., Rodrigues N.B., Phan L., Chen-Li D. (2020). The association between adverse childhood experiences and inflammation in patients with major depressive disorder: A systematic review. J. Affect. Disord..

[38] MacDonald K., Thomas M.L., Sciolla A.F., Schneider B., Pappas K., Bleijenberg G., Bohus M., Bekh B., Carpenter L., Carr A. (2016). Minimization of childhood maltreatment is common and consequential: Results from a large, multinational sample using the Childhood Trauma Questionnaire. PLoS ONE.

[39] Sharma V., Khan M., Smith A. (2005). A closer look at treatment resistant depression: Is it due to a bipolar diathesis?. J. Affect. Disord..

[40] Janiri D., Sani G., Danese E., Simonetti A., Ambrosi E., Angeletti G., Erbuto D., Caltagirone C., Girardi P., Spalletta G. (2015). Childhood traumatic experiences of patients with bipolar disorder type I and type II. J. Affect. Disord..

[41] Bardi F., Pinto M., Simonetti A., Di Nicola M., Mazza M., Manfredi G., Kotzalidis G.D., Sani G., Janiri D. (2026). Lifetime mixed depression and childhood trauma in individuals with bipolar disorders. Acta Psychiatr. Scand..

[42] Janiri D., Simonetti A., Luciano M., Montanari S., Bernardi E., Carrà G., Fiorillo A., Sani G. (2024). Type of cycle, temperament and childhood trauma are associated with lithium response in patients with bipolar disorders. Int. J. Bipolar Disord..

[43] Fekadu A., Wooderson S.C., Markopoulou K., Cleare A.J. (2009). The Maudsley Staging Method for treatment-resistant depression: Prediction of longer-term outcome and persistence of symptoms. J. Clin. Psychiatry.

