# Supplementary material for: Dimension-Specific Childhood Maltreatment and Cross-Sectional Markers of Illness Burden in Treatment-Resistant Depression: A Retrospective Single-Center Study
Source: J Clin Med. 2026 Oct 21;15(20):8182. doi: 10.3390/jcm15208182 (PMC13706073; doi:10.3390/jcm15208182)
Supplement: Supplementary file 1 [file jcm-15-08182-s001.zip › jcm-4582989-supplementary.pdf]

Supplementary Materials

Supplementary tables provide complete reporting of the primary model family and reviewer-requested sensitivity, missing-data, diagnostic, distributional, nonlinearity, and influence analyses. Primary and exploratory families are labeled explicitly.

Table S1A. Complete primary model family: ongoing major depressive episode duration at assessment.

CTQ exposure	Increment	N	β (robust SE)	Adjusted effect	95% CI	p; global q
CTQ total	+10 points	104	0.0049 (0.1009)	+0.5%	-17.7% to +22.8%	0.962; 0.979
Trauma load	+1 dimension	104	-0.0156 (0.0749)	-1.6%	-15.2% to +14.2%	0.835; 0.979
Emotional abuse	+5 points	104	0.1136 (0.0829)	+12.0%	-5.0% to +32.1%	0.173; 0.486
Physical abuse	+5 points	104	0.4076 (0.0969)	+50.3%	+24.0% to +82.2%	<0.001; <0.001
Sexual abuse	+5 points	104	0.4687 (0.1178)	+59.8%	+26.5% to +101.9%	<0.001; 0.001
Emotional neglect	+5 points	104	-0.2890 (0.0927)	-25.1%	-37.7% to -10.0%	0.002; 0.013
Physical neglect	+5 points	104	-0.0704 (0.1615)	-6.8%	-32.4% to +28.4%	0.664; 0.979

Table S1B. Complete primary model family: illness duration.

CTQ exposure	Increment	N	β (robust SE)	Adjusted effect	95% CI	p; global q
CTQ total	+10 points	99	-0.1472 (0.7926)	-0.15 y	-1.72 to +1.43	0.853; 0.979
Trauma load	+1 dimension	99	0.0251 (0.9057)	+0.03 y	-1.77 to +1.82	0.978; 0.979
Emotional abuse	+5 points	99	0.3355 (0.9387)	+0.34 y	-1.53 to +2.20	0.722; 0.979
Physical abuse	+5 points	99	0.6021 (1.5778)	+0.60 y	-2.53 to +3.73	0.704; 0.979
Sexual abuse	+5 points	99	0.9399 (1.8205)	+0.94 y	-2.67 to +4.55	0.607; 0.979
Emotional neglect	+5 points	99	0.0843 (1.0246)	+0.08 y	-1.95 to +2.12	0.935; 0.979
Physical neglect	+5 points	99	-2.9373 (1.6043)	-2.94 y	-6.12 to +0.25	0.070; 0.281

Table S1C. Complete primary model family: number of previous major depressive episodes.

CTQ exposure	Increment	N	β (robust SE)	Adjusted effect	95% CI	p; global q
CTQ total	+10 points	93	-0.0414 (0.0611)	IRR 0.96	0.85–1.08	0.497; 0.979
Trauma load	+1 dimension	93	0.0019 (0.0725)	IRR 1.00	0.87–1.15	0.979; 0.979
Emotional abuse	+5 points	93	0.0432 (0.0701)	IRR 1.04	0.91–1.20	0.538; 0.979

CTQ exposure	Increment	N	β (robust SE)	Adjusted effect	95% CI	p; global q
Physical abuse	+5 points	93	0.0668 (0.1644)	IRR 1.07	0.77–1.48	0.684; 0.979
Sexual abuse	+5 points	93	0.0395 (0.2142)	IRR 1.04	0.68–1.58	0.854; 0.979
Emotional neglect	+5 points	93	-0.0925 (0.0655)	IRR 0.91	0.80–1.04	0.158; 0.486
Physical neglect	+5 points	93	-0.2525 (0.1153)	IRR 0.78	0.62–0.97	0.029; 0.133

Table S1D. Complete primary model family: lifetime failed antidepressant trials.

CTQ exposure	Increment	N	β (robust SE)	Adjusted effect	95% CI	p; global q
CTQ total	+10 points	104	-0.0090 (0.0694)	IRR 0.99	0.87–1.14	0.897; 0.979
Trauma load	+1 dimension	104	-0.0124 (0.0542)	IRR 0.99	0.89–1.10	0.819; 0.979
Emotional abuse	+5 points	104	0.0639 (0.0530)	IRR 1.07	0.96–1.18	0.229; 0.582
Physical abuse	+5 points	104	0.1796 (0.0432)	IRR 1.20	1.10–1.30	<0.001; <0.001
Sexual abuse	+5 points	104	0.1520 (0.1089)	IRR 1.16	0.94–1.44	0.163; 0.486
Emotional neglect	+5 points	104	-0.1609 (0.0512)	IRR 0.85	0.77–0.94	0.002; 0.012

CTQ exposure	Increment	N	β (robust SE)	Adjusted effect	95% CI	p; global q
Physical neglect	+5 points	104	-0.0842 (0.1206)	IRR 0.92	0.73–1.16	0.485; 0.979

Primary models adjust for current age, sex, and internal extraction block; extraction block was inestimable for previous-MDE models because that outcome was present only in the principal block. β is the coefficient on the model scale and SE is the HC3 or sandwich-robust standard error. Global q values are Benjamini–Hochberg adjusted across all 28 primary tests. Inferential results were regenerated during the first revision from the same corrected dataset using HC3 covariance and residual-degrees-of-freedom t tests for linear models, and leverage-adjusted sandwich covariance and normal-reference Wald tests for count models. This revised inferential workflow changed confidence intervals, p values, and q values even where rounded point estimates were unchanged. For sexual abuse and failed trials, the IRR remained 1.16, while the 95% CI changed from 1.01–1.34 in the first submission to 0.94–1.44 in the revised analysis (p=0.163; global q=0.486).

Table S2. Per-standard-deviation estimates for all 28 primary exposure–outcome combinations.

Exposure	SD	Outcome	N	Adjusted effect	95% CI	p	q
CTQ total	14.59	Elapsed ongoing-MDE duration	104	+0.7%	-24.8% to +34.9%	0.962	0.979
Trauma load	1.33	Elapsed ongoing-MDE duration	104	-2.1%	-19.7% to +19.4%	0.835	0.979
Emotional abuse	4.95	Elapsed ongoing-MDE duration	104	+11.9%	-4.9% to +31.7%	0.173	0.486
Physical abuse	3.52	Elapsed ongoing-MDE duration	104	+33.2%	+16.4% to +52.6%	<0.001	<0.001
Sexual abuse	2.41	Elapsed ongoing-MDE duration	104	+25.4%	+12.0% to +40.3%	<0.001	0.001
Emotional neglect	5.65	Elapsed ongoing-MDE duration	104	-27.8%	-41.4% to -11.2%	0.002	0.013
Physical neglect	4.36	Elapsed ongoing-MDE duration	104	-6.0%	-28.9% to +24.4%	0.664	0.979
CTQ total	14.59	Illness duration	99	-0.21 y	-2.51 to +2.08	0.853	0.979
Trauma load	1.33	Illness duration	99	+0.03 y	-2.36 to +2.43	0.978	0.979
Emotional abuse	4.95	Illness duration	99	+0.33 y	-1.51 to +2.18	0.722	0.979
Physical abuse	3.52	Illness duration	99	+0.42 y	-1.78 to +2.63	0.704	0.979
Sexual abuse	2.41	Illness duration	99	+0.45 y	-1.29 to +2.20	0.607	0.979
Emotional neglect	5.65	Illness duration	99	+0.10 y	-2.20 to +2.39	0.935	0.979
Physical neglect	4.36	Illness duration	99	-2.56 y	-5.34 to +0.22	0.070	0.281
CTQ total	14.59	Previous MDEs	93	IRR 0.94	0.79–1.12	0.497	0.979
Trauma load	1.33	Previous MDEs	93	IRR 1.00	0.83–1.21	0.979	0.979

Exposure	SD	Outcome	N	Adjusted effect	95% CI	p	q
Emotional abuse	4.95	Previous MDEs	93	IRR 1.04	0.91–1.20	0.538	0.979
Physical abuse	3.52	Previous MDEs	93	IRR 1.05	0.84–1.32	0.684	0.979
Sexual abuse	2.41	Previous MDEs	93	IRR 1.02	0.83–1.25	0.854	0.979
Emotional neglect	5.65	Previous MDEs	93	IRR 0.90	0.78–1.04	0.158	0.486
Physical neglect	4.36	Previous MDEs	93	IRR 0.80	0.66–0.98	0.029	0.133
CTQ total	14.59	Lifetime failed antidepressant trials	104	IRR 0.99	0.81–1.20	0.897	0.979
Trauma load	1.33	Lifetime failed antidepressant trials	104	IRR 0.98	0.85–1.13	0.819	0.979
Emotional abuse	4.95	Lifetime failed antidepressant trials	104	IRR 1.07	0.96–1.18	0.229	0.582
Physical abuse	3.52	Lifetime failed antidepressant trials	104	IRR 1.13	1.07–1.20	<0.001	<0.001
Sexual abuse	2.41	Lifetime failed antidepressant trials	104	IRR 1.08	0.97–1.19	0.163	0.486
Emotional neglect	5.65	Lifetime failed antidepressant trials	104	IRR 0.83	0.74–0.93	0.002	0.012
Physical neglect	4.36	Lifetime failed antidepressant trials	104	IRR 0.93	0.76–1.14	0.485	0.979

Table S3. Clinical-cutoff sensitivity analyses for all four outcomes (exploratory family of 20 tests).

Dimension	Cutoff	Exposed N	Outcome	Effect	95% CI	p; q
Emotional abuse	≥13	23	Elapsed ongoing-MDE duration	+27.3%	-9.2% to +78.5%	0.160; 0.355
Emotional abuse	≥13	23	Illness duration	+2.49 y	-2.45 to +7.44	0.320; 0.492
Emotional abuse	≥13	23	Previous MDEs	IRR 1.26	0.85–1.85	0.246; 0.448
Emotional abuse	≥13	23	Lifetime failed antidepressant trials	IRR 1.15	0.90–1.46	0.270; 0.449
Physical abuse	≥10	13	Elapsed ongoing-MDE duration	+100.9%	+26.6% to +218.8%	0.003; 0.033
Physical abuse	≥10	13	Illness duration	+0.88 y	-5.72 to +7.48	0.792; 0.833

Dimension	Cutoff	Exposed N	Outcome	Effect	95% CI	p; q
Physical abuse	≥10	13	Previous MDEs	IRR 1.19	0.64–2.25	0.581; 0.684
Physical abuse	≥10	13	Lifetime failed antidepressant trials	IRR 1.46	1.12–1.90	0.005; 0.033
Sexual abuse	≥8	24	Elapsed ongoing-MDE duration	+67.4%	+2.9% to +172.3%	0.038; 0.127
Sexual abuse	≥8	24	Illness duration	+0.72 y	-6.07 to +7.51	0.833; 0.833
Sexual abuse	≥8	24	Previous MDEs	IRR 1.28	0.69–2.39	0.433; 0.619
Sexual abuse	≥8	24	Lifetime failed antidepressant trials	IRR 1.11	0.82–1.49	0.512; 0.655
Emotional neglect	≥15	59	Elapsed ongoing-MDE duration	-39.0%	-55.8% to -15.7%	0.003; 0.033
Emotional neglect	≥15	59	Illness duration	+1.44 y	-3.03 to +5.90	0.524; 0.655
Emotional neglect	≥15	59	Previous MDEs	IRR 0.94	0.67–1.31	0.704; 0.782
Emotional neglect	≥15	59	Lifetime failed antidepressant trials	IRR 0.76	0.61–0.94	0.014; 0.068
Physical neglect	≥10	49	Elapsed ongoing-MDE duration	-31.9%	-54.6% to +2.2%	0.063; 0.181
Physical neglect	≥10	49	Illness duration	-4.51 y	-9.52 to +0.50	0.077; 0.193
Physical neglect	≥10	49	Previous MDEs	IRR 0.71	0.51–0.98	0.036; 0.127
Physical neglect	≥10	49	Lifetime failed antidepressant trials	IRR 0.85	0.65–1.11	0.224; 0.447

Abbreviations and FDR families are defined beneath each table. Primary q values refer only to the 28-test primary family; secondary q values are adjusted within the family named in the table and are exploratory.

Table S4A. Comparison of included (N=104) and excluded (N=30) source records.

**Variable**	**Included**	**Excluded**	**SMD**	**p**
Age, years	54.95 (13.45); median 57.00 (n=104)	57.33 (11.02); median 56.50 (n=30)	-0.18	0.644
Elapsed ongoing-MDE duration	24.00 (17.54); median 18.00 (n=104)	17.63 (14.03); median 12.00 (n=27)	0.38	0.081
Age at first MDE, years	36.79 (14.68); median 35.00 (n=99)	39.20 (11.58); median 39.50 (n=30)	-0.17	0.244
Previous MDEs	2.63 (2.15); median 2.00 (n=93)	3.52 (1.99); median 3.00 (n=27)	-0.42	0.016
Lifetime failed antidepressant trials	4.09 (2.22); median 4.00 (n=104)	2.33 (1.12); median 2.00 (n=30)	0.86	<0.001

Variable	Included	Excluded	SMD	p
Sex	Male 60; female 44 (n=104)	Male 17; female 13 (n=30)	—	0.920
Marital status	Single 20; married 73; widowed 11 (n=104)	Single 13; married 17; widowed 0 (n=30)	—	0.010
Any recorded psychiatric comorbidity	No 63; yes 40; missing 1 (n=104)	No 1; yes 29 (n=30)	—	<0.001
Internal extraction block	Principal 93; secondary 11 (n=104)	Principal 27; secondary 3 (n=30)	—	0.927

Values are mean (SD) and median, or counts. Denominators are outcome-specific; the psychiatric-comorbidity comparison has one missing included record. SMD, pooled standardized mean difference for continuous variables. Mann–Whitney tests were used for continuous variables; χ^2 tests for categorical variables. These descriptive comparisons have unadjusted p values and are not confirmatory hypothesis tests. Recorded comorbidity was derived from numeric codes: 0=no recorded comorbidity; nonzero=recorded comorbidity; a missing code remained missing. Rechecking the original numeric entries confirmed the tabulated 40/103 included and 29/30 excluded records; this coding check is not an independent diagnostic audit. All 27 out-of-range CTQ records belonged to the principal extraction block (120 source records: 93 included, 27 excluded); the three records excluded for missing clinical CTQ items belonged to the secondary export (14 source records: 11 included, three excluded). Block membership does not identify the specific import event that caused the errors. Non-CTQ fields in excluded records, including comorbidity and failed-trial counts, were not independently verified against original clinical records. These comparisons are therefore descriptive contrasts of recorded entries and may reflect clinical differences, additional coding errors, or both.

Table S4B. Participants with versus without the two secondary clinical outcomes.

Outcome	Comparison variable	Observed N	Observed mean/%	Missing N	Missing mean/%	p
Illness duration	age	99	55.08	5	52.40	0.958
Illness duration	Female, %	99	43.4%	5	20.0%	0.393
Illness duration	Secondary extraction block, %	99	6.1%	5	100.0%	<0.001
Illness duration	CTQ total	99	46.59	5	64.40	0.001
Illness duration	Physical abuse	99	6.93	5	8.20	0.013
Illness duration	Sexual abuse	99	6.01	5	9.00	<0.001
Illness duration	Emotional neglect	99	14.31	5	19.80	0.018
Illness duration	Lifetime failed antidepressant trials	99	4.18	5	2.20	0.018
Illness duration	Elapsed ongoing-MDE duration	99	23.63	5	31.40	0.617
Previous MDEs	age	93	54.84	11	55.91	0.593
Previous MDEs	Female, %	93	43.0%	11	36.4%	0.756
Previous MDEs	Secondary extraction block, %	93	0.0%	11	100.0%	<0.001
Previous MDEs	CTQ total	93	45.25	11	66.00	<0.001

Outcome	Comparison variable	Observed N	Observed mean/%	Missing N	Missing mean/%	p
Previous MDEs	Physical abuse	93	6.74	11	9.09	<0.001
Previous MDEs	Sexual abuse	93	5.77	11	9.36	<0.001
Previous MDEs	Emotional neglect	93	14.04	11	19.09	0.002
Previous MDEs	Lifetime failed antidepressant trials	93	4.18	11	3.27	0.151
Previous MDEs	Elapsed ongoing-MDE duration	93	23.84	11	25.36	0.940

Descriptive unadjusted comparisons: Mann–Whitney tests for continuous variables and Fisher exact tests for binary variables. Five illness-duration and 11 previous-MDE values were missing, all in the secondary extraction block. Missingness was associated with CTQ exposure, so these complete-outcome analyses may not represent the full cohort.

Table S5A. Continuous-variable and CTQ exposure distributions.

Variable	N	Mean	SD	Median	IQR	Range
Age, years	104	54.95	13.45	57.00	48.00–64.25	20.00–81.00
Age at first MDE, years	99	36.79	14.68	35.00	25.50–46.50	12.00–76.00
Illness duration	99	18.29	12.08	18.00	7.00–27.50	0.00–44.00
Elapsed ongoing-MDE duration	104	24.00	17.54	18.00	12.00–36.00	1.00–84.00
Previous MDEs	93	2.63	2.15	2.00	1.00–3.00	0.00–10.00
Lifetime failed antidepressant trials	104	4.09	2.22	4.00	2.00–5.00	1.00–12.00
CTQ total	104	47.44	14.59	49.00	36.00–57.00	25.00–115.00
Trauma load	104	1.62	1.33	2.00	0.00–3.00	0.00–5.00
Emotional abuse	104	9.49	4.95	8.00	5.00–12.00	5.00–25.00
Physical abuse	104	6.99	3.52	5.00	5.00–7.00	5.00–23.00
Sexual abuse	104	6.15	2.41	5.00	5.00–6.00	5.00–22.00
Emotional neglect	104	14.58	5.65	15.00	10.00–19.00	5.00–25.00
Physical neglect	104	10.23	4.36	9.00	7.00–13.00	5.00–25.00
Minimization/Denial score	104	0.31	0.79	0.00	0.00–0.00	0.00–3.00

Table S5B. Spearman correlations among CTQ subscales.

Subscale	Emotional abuse	Physical abuse	Sexual abuse	Emotional neglect	Physical neglect
Emotional abuse	1.00	0.55	0.37	0.26	0.27
Physical abuse	0.55	1.00	0.43	0.15	0.35
Sexual abuse	0.37	0.43	1.00	0.22	0.39
Emotional neglect	0.26	0.15	0.22	1.00	0.64
Physical neglect	0.27	0.35	0.39	0.64	1.00

Table S5C. Collinearity diagnostics for the mutually adjusted five-subscale model.

Subscale	VIF	Tolerance
Emotional abuse	1.48	0.68
Physical abuse	1.73	0.58
Sexual abuse	1.84	0.54
Emotional neglect	1.80	0.56
Physical neglect	3.08	0.32

Table S6A. Count-outcome category frequencies.

Outcome	Category	N	%
Previous MDEs	0	9	9.7
Previous MDEs	1	21	22.6
Previous MDEs	2	25	26.9
Previous MDEs	3	16	17.2
Previous MDEs	4	6	6.5
Previous MDEs	5	9	9.7
Previous MDEs	6	2	2.2
Previous MDEs	7	1	1.1
Previous MDEs	8	1	1.1

Outcome	Category	N	%
Previous MDEs	10	3	3.2
Lifetime failed antidepressant trials	1	1	1.0
Lifetime failed antidepressant trials	2	32	30.8
Lifetime failed antidepressant trials	3	13	12.5
Lifetime failed antidepressant trials	4	25	24.0
Lifetime failed antidepressant trials	5	11	10.6
Lifetime failed antidepressant trials	6	8	7.7
Lifetime failed antidepressant trials	7	5	4.8
Lifetime failed antidepressant trials	8	5	4.8
Lifetime failed antidepressant trials	9	1	1.0
Lifetime failed antidepressant trials	10	1	1.0
Lifetime failed antidepressant trials	12	2	1.9

Table S6B. Poisson model diagnostics for all primary count models.

Outcome	Exposure	N	Pearson dispersion	Deviance ratio	AIC
Previous MDEs	CTQ total	93	1.69	1.65	388.2
Previous MDEs	Trauma load	93	1.70	1.65	388.9
Previous MDEs	Emotional abuse	93	1.71	1.65	388.4
Previous MDEs	Physical abuse	93	1.69	1.65	388.3
Previous MDEs	Sexual abuse	93	1.70	1.65	388.8
Previous MDEs	Emotional neglect	93	1.66	1.62	386.3
Previous MDEs	Physical neglect	93	1.58	1.58	382.2
Lifetime failed antidepressant trials	CTQ total	104	1.21	1.10	448.1
Lifetime failed antidepressant trials	Trauma load	104	1.21	1.10	448.0
Lifetime failed antidepressant trials	Emotional abuse	104	1.21	1.08	446.4

Outcome	Exposure	N	Pearson dispersion	Deviance ratio	AIC
Lifetime failed antidepressant trials	Physical abuse	104	1.15	1.02	440.0
Lifetime failed antidepressant trials	Sexual abuse	104	1.20	1.08	445.9
Lifetime failed antidepressant trials	Emotional neglect	104	1.07	0.97	435.3
Lifetime failed antidepressant trials	Physical neglect	104	1.20	1.09	446.8

Table S6C. Ordinal failed-trial sensitivity and proportional-odds diagnostics (n=103; categories 2, 3, 4, 5, ≥6).

Exposure	Common OR	95% CI	p; q	Exposure Wald p	Omnibus Wald p
CTQ total	0.95	0.70–1.27	0.719; 0.719	0.346	<0.001
Trauma load	0.90	0.66–1.22	0.497; 0.580	0.179	<0.001
Emotional abuse	1.36	0.94–1.95	0.102; 0.157	0.608	<0.001
Physical abuse	2.94	1.53–5.63	0.001; 0.004	0.307	<0.001
Sexual abuse	2.38	0.82–6.97	0.112; 0.157	0.501	<0.001
Emotional neglect	0.49	0.35–0.70	<0.001; <0.001	0.991	<0.001
Physical neglect	0.59	0.33–1.06	0.078; 0.157	0.132	<0.001

The single count of 1 was excluded. Counts ≥6 were pooled because the secondary block had no observations ≥7, producing complete separation in the highest threshold diagnostic. Model p values are Wald tests; q values use a seven-exposure exploratory family. Assumption tests compare slopes at ≥3, ≥4, ≥5, and ≥6 using their joint participant-level sandwich covariance (exposure df=3; omnibus df=12). The omnibus assumption failed in all seven models; common ORs are therefore exploratory, not confirmatory evidence. Assumption-test p values are unadjusted.

Table S6D. Residual, heteroskedasticity, and leverage diagnostics for the primary log-duration models.

Exposure	N	Shapiro W	Shapiro p	Breusch–Pagan LM	BP p	Maximum leverage
CTQ total	104	0.924	<0.001	10.40	0.034	0.297
Trauma load	104	0.926	<0.001	9.28	0.054	0.164
Emotional abuse	104	0.932	<0.001	9.52	0.049	0.167
Physical abuse	104	0.925	<0.001	9.61	0.047	0.242
Sexual abuse	104	0.929	<0.001	9.65	0.047	0.573
Emotional neglect	104	0.962	0.004	19.35	<0.001	0.161
Physical neglect	104	0.931	<0.001	12.49	0.014	0.165

Diagnostic p values are descriptive and unadjusted. HC3 errors address heteroskedasticity and high leverage; bootstrap analyses further assess inference under non-normal residuals.

Table S6E. Exposure slopes from the cumulative binary threshold diagnostics.

Exposure	Failed-trial threshold	Events	Adjusted OR	95% CI	SE of log OR
CTQ total	≥3	71	0.85	0.59–1.24	0.1904
CTQ total	≥4	58	0.88	0.59–1.31	0.2044
CTQ total	≥5	33	1.03	0.67–1.58	0.2178
CTQ total	≥6	22	1.18	0.71–1.95	0.2581
Trauma load	≥3	71	0.74	0.52–1.07	0.1842
Trauma load	≥4	58	0.79	0.54–1.14	0.1891
Trauma load	≥5	33	1.07	0.73–1.59	0.2008
Trauma load	≥6	22	1.21	0.74–1.97	0.2494
Emotional abuse	≥3	71	1.21	0.77–1.89	0.2292
Emotional abuse	≥4	58	1.20	0.78–1.82	0.2158
Emotional abuse	≥5	33	1.45	0.93–2.25	0.2263
Emotional abuse	≥6	22	1.66	1.02–2.70	0.2490
Physical abuse	≥3	71	4.02	1.16–13.96	0.6355
Physical abuse	≥4	58	2.36	1.02–5.45	0.4281
Physical abuse	≥5	33	2.54	1.20–5.36	0.3815
Physical abuse	≥6	22	3.48	1.58–7.70	0.4043
Sexual abuse	≥3	71	1.51	0.32–7.14	0.7945
Sexual abuse	≥4	58	2.04	0.41–10.21	0.8212
Sexual abuse	≥5	33	2.06	0.65–6.60	0.5932
Sexual abuse	≥6	22	3.07	0.77–12.27	0.7068
Emotional neglect	≥3	71	0.49	0.31–0.78	0.2375
Emotional neglect	≥4	58	0.47	0.29–0.77	0.2438

Exposure	Failed-trial threshold	Events	Adjusted OR	95% CI	SE of log OR
Emotional neglect	≥5	33	0.48	0.29–0.81	0.2608
Emotional neglect	≥6	22	0.46	0.26–0.81	0.2929
Physical neglect	≥3	71	0.45	0.20–1.00	0.4095
Physical neglect	≥4	58	0.61	0.30–1.26	0.3706
Physical neglect	≥5	33	0.88	0.40–1.92	0.3967
Physical neglect	≥6	22	0.92	0.33–2.55	0.5193

All threshold models use n=103 and adjust for age, sex, and extraction block. Covariance-aware comparisons of these dependent threshold fits are in Table S6C. Estimates are per the primary CTQ increments and are diagnostic rather than separate efficacy claims.

Table S7A. Predictive-mean-matching multiple imputation for the systematic Item 28 omission (50 datasets; 12 affected models).

Exposure	Outcome	m	Effect	95% CI	p; q
CTQ total	Elapsed ongoing-MDE duration	50	+0.5%	-17.6% to +22.6%	0.960; 0.993
Emotional neglect	Elapsed ongoing-MDE duration	50	-25.0%	-37.5% to -9.9%	0.002; 0.012
Trauma load	Elapsed ongoing-MDE duration	50	-1.3%	-14.7% to +14.1%	0.856; 0.993
CTQ total	Lifetime failed antidepressant trials	50	IRR 0.99	0.86–1.14	0.892; 0.993
Emotional neglect	Lifetime failed antidepressant trials	50	IRR 0.85	0.77–0.94	0.002; 0.012
Trauma load	Lifetime failed antidepressant trials	50	IRR 0.99	0.89–1.10	0.860; 0.993
CTQ total	Illness duration	50	-0.14 y	-1.70 to +1.41	0.858; 0.993
Emotional neglect	Illness duration	50	+0.10 y	-1.91 to +2.11	0.923; 0.993
Trauma load	Illness duration	50	+0.01 y	-1.76 to +1.77	0.993; 0.993
CTQ total	Previous MDEs	50	IRR 0.96	0.85–1.08	0.497; 0.993
Emotional neglect	Previous MDEs	50	IRR 0.91	0.80–1.04	0.158; 0.632
Trauma load	Previous MDEs	50	IRR 1.00	0.87–1.15	0.979; 0.993

PMM used five nearest observed donors per missing response, stochastic regression-parameter/variance draws, and Rubin pooling across 50 datasets (seed 20261007). Predictors were the other 24 clinical CTQ items, age, sex, log ongoing-MDE duration, and failed-trial count; observed donor values retained the 1–5 range. Item 28 was absent throughout the secondary block, so imputation cannot estimate a residual block effect and assumes conditional transportability from the observed block. This is an exploratory conditional-MAR sensitivity, not proof of random missingness. q values use the 12 affected exposure–outcome models. Previous-MDE models are unchanged because their 93 records had observed Item 28.

Table S7B. Complete-case analysis excluding all Item 28 replacements.

Exposure	Outcome	N	β (SE)	Effect	95% CI	p; q
CTQ total	Elapsed ongoing-MDE duration	93	-0.0022 (0.1093)	-0.2%	-19.7% to +24.0%	0.984; 0.997
Trauma load	Elapsed ongoing-MDE duration	93	-0.0053 (0.0767)	-0.5%	-14.6% to +15.8%	0.945; 0.997
Emotional abuse	Elapsed ongoing-MDE duration	93	0.1197 (0.0848)	+12.7%	-4.8% to +33.4%	0.161; 0.502
Physical abuse	Elapsed ongoing-MDE duration	93	0.4060 (0.1051)	+50.1%	+21.8% to +84.9%	<0.001; 0.003
Sexual abuse	Elapsed ongoing-MDE duration	93	0.4948 (0.1182)	+64.0%	+29.7% to +107.4%	<0.001; 0.002
Emotional neglect	Elapsed ongoing-MDE duration	93	-0.2931 (0.0927)	-25.4%	-38.0% to -10.3%	0.002; 0.015
Physical neglect	Elapsed ongoing-MDE duration	93	-0.1142 (0.1738)	-10.8%	-36.8% to +26.0%	0.513; 0.967
CTQ total	Illness duration	93	-0.1775 (0.8101)	-0.18 y	-1.79 to +1.43	0.827; 0.997
Trauma load	Illness duration	93	0.0037 (0.9144)	+0.00 y	-1.81 to +1.82	0.997; 0.997
Emotional abuse	Illness duration	93	0.3752 (0.9394)	+0.38 y	-1.49 to +2.24	0.691; 0.967
Physical abuse	Illness duration	93	0.6865 (1.6637)	+0.69 y	-2.62 to +3.99	0.681; 0.967
Sexual abuse	Illness duration	93	0.8805 (1.8143)	+0.88 y	-2.72 to +4.49	0.629; 0.967
Emotional neglect	Illness duration	93	0.0335 (1.0411)	+0.03 y	-2.04 to +2.10	0.974; 0.997
Physical neglect	Illness duration	93	-3.2302 (1.6812)	-3.23 y	-6.57 to +0.11	0.058; 0.232
CTQ total	Previous MDEs	93	-0.0414 (0.0611)	IRR 0.96	0.85–1.08	0.497; 0.967
Trauma load	Previous MDEs	93	0.0019 (0.0725)	IRR 1.00	0.87–1.15	0.979; 0.997
Emotional abuse	Previous MDEs	93	0.0432 (0.0701)	IRR 1.04	0.91–1.20	0.538; 0.967
Physical abuse	Previous MDEs	93	0.0668 (0.1644)	IRR 1.07	0.77–1.48	0.684; 0.967
Sexual abuse	Previous MDEs	93	0.0395 (0.2142)	IRR 1.04	0.68–1.58	0.854; 0.997
Emotional neglect	Previous MDEs	93	-0.0925 (0.0655)	IRR 0.91	0.80–1.04	0.158; 0.502
Physical neglect	Previous MDEs	93	-0.2525 (0.1153)	IRR 0.78	0.62–0.97	0.029; 0.133
CTQ total	Lifetime failed antidepressant trials	93	-0.0185 (0.0753)	IRR 0.98	0.85–1.14	0.806; 0.997
Trauma load	Lifetime failed antidepressant trials	93	-0.0268 (0.0560)	IRR 0.97	0.87–1.09	0.633; 0.967

Exposure	Outcome	N	β (SE)	Effect	95% CI	p; q
Emotional abuse	Lifetime failed antidepressant trials	93	0.0528 (0.0552)	IRR 1.05	0.95–1.17	0.339; 0.862
Physical abuse	Lifetime failed antidepressant trials	93	0.1571 (0.0478)	IRR 1.17	1.07–1.29	0.001; 0.010
Sexual abuse	Lifetime failed antidepressant trials	93	0.1465 (0.1297)	IRR 1.16	0.90–1.49	0.259; 0.724
Emotional neglect	Lifetime failed antidepressant trials	93	-0.1532 (0.0522)	IRR 0.86	0.77–0.95	0.003; 0.019
Physical neglect	Lifetime failed antidepressant trials	93	-0.1101 (0.1287)	IRR 0.90	0.70–1.15	0.392; 0.916

Exploratory 28-test family, with within-family BH q values. Primary models and exposure increments are retained; β and SE are on the model scale.

Table S8. Full models restricted to the principal internal extraction block.

Exposure	Outcome	N	Effect	95% CI	p; q
CTQ total	Elapsed ongoing-MDE duration	93	-0.2%	-19.7% to +24.0%	0.984; 0.997
Trauma load	Elapsed ongoing-MDE duration	93	-0.5%	-14.6% to +15.8%	0.945; 0.997
Emotional abuse	Elapsed ongoing-MDE duration	93	+12.7%	-4.8% to +33.4%	0.161; 0.502
Physical abuse	Elapsed ongoing-MDE duration	93	+50.1%	+21.8% to +84.9%	<0.001; 0.003
Sexual abuse	Elapsed ongoing-MDE duration	93	+64.0%	+29.7% to +107.4%	<0.001; 0.002
Emotional neglect	Elapsed ongoing-MDE duration	93	-25.4%	-38.0% to -10.3%	0.002; 0.015
Physical neglect	Elapsed ongoing-MDE duration	93	-10.8%	-36.8% to +26.0%	0.513; 0.967
CTQ total	Illness duration	93	-0.18 y	-1.79 to +1.43	0.827; 0.997
Trauma load	Illness duration	93	+0.00 y	-1.81 to +1.82	0.997; 0.997
Emotional abuse	Illness duration	93	+0.38 y	-1.49 to +2.24	0.691; 0.967
Physical abuse	Illness duration	93	+0.69 y	-2.62 to +3.99	0.681; 0.967
Sexual abuse	Illness duration	93	+0.88 y	-2.72 to +4.49	0.629; 0.967
Emotional neglect	Illness duration	93	+0.03 y	-2.04 to +2.10	0.974; 0.997
Physical neglect	Illness duration	93	-3.23 y	-6.57 to +0.11	0.058; 0.232
CTQ total	Previous MDEs	93	IRR 0.96	0.85–1.08	0.497; 0.967
Trauma load	Previous MDEs	93	IRR 1.00	0.87–1.15	0.979; 0.997

Exposure	Outcome	N	Effect	95% CI	p; q
Emotional abuse	Previous MDEs	93	IRR 1.04	0.91–1.20	0.538; 0.967
Physical abuse	Previous MDEs	93	IRR 1.07	0.77–1.48	0.684; 0.967
Sexual abuse	Previous MDEs	93	IRR 1.04	0.68–1.58	0.854; 0.997
Emotional neglect	Previous MDEs	93	IRR 0.91	0.80–1.04	0.158; 0.502
Physical neglect	Previous MDEs	93	IRR 0.78	0.62–0.97	0.029; 0.133
CTQ total	Lifetime failed antidepressant trials	93	IRR 0.98	0.85–1.14	0.806; 0.997
Trauma load	Lifetime failed antidepressant trials	93	IRR 0.97	0.87–1.09	0.633; 0.967
Emotional abuse	Lifetime failed antidepressant trials	93	IRR 1.05	0.95–1.17	0.339; 0.862
Physical abuse	Lifetime failed antidepressant trials	93	IRR 1.17	1.07–1.29	0.001; 0.010
Sexual abuse	Lifetime failed antidepressant trials	93	IRR 1.16	0.90–1.49	0.259; 0.724
Emotional neglect	Lifetime failed antidepressant trials	93	IRR 0.86	0.77–0.95	0.003; 0.019
Physical neglect	Lifetime failed antidepressant trials	93	IRR 0.90	0.70–1.15	0.392; 0.916

Exploratory 28-test family with within-family BH q values. Extraction-block adjustment is unnecessary after restriction; models retain age and sex.

Table S9. Mutually adjusted five-subscale models (exploratory family of 20 tests).

Subscale	Outcome	N	β (SE)	Effect	95% CI	p; q
Emotional abuse	Elapsed ongoing-MDE duration	104	0.0287 (0.0747)	+2.9%	-11.3% to +19.4%	0.701; 0.814
Physical abuse	Elapsed ongoing-MDE duration	104	0.3248 (0.1410)	+38.4%	+4.6% to +83.1%	0.023; 0.117
Sexual abuse	Elapsed ongoing-MDE duration	104	0.2665 (0.1622)	+30.5%	-5.4% to +80.1%	0.104; 0.259
Emotional neglect	Elapsed ongoing-MDE duration	104	-0.3400 (0.1005)	-28.8%	-41.7% to -13.1%	0.001; 0.021
Physical neglect	Elapsed ongoing-MDE duration	104	0.0597 (0.1297)	+6.2%	-17.9% to +37.3%	0.646; 0.814
Emotional abuse	Illness duration	99	-0.4716 (1.1902)	-0.47 y	-2.84 to +1.89	0.693; 0.814
Physical abuse	Illness duration	99	2.1172 (2.1236)	+2.12 y	-2.10 to +6.34	0.321; 0.714
Sexual abuse	Illness duration	99	2.3982 (2.6629)	+2.40 y	-2.89 to +7.69	0.370; 0.740
Emotional neglect	Illness duration	99	2.1627 (1.2504)	+2.16 y	-0.32 to +4.65	0.087; 0.249

Subscale	Outcome	N	β (SE)	Effect	95% CI	p; q
Physical neglect	Illness duration	99	-6.0488 (1.9450)	-6.05 y	-9.91 to -2.18	0.003; 0.022
Emotional abuse	Previous MDEs	93	0.0351 (0.0902)	IRR 1.04	0.87–1.24	0.697; 0.814
Physical abuse	Previous MDEs	93	0.1174 (0.2253)	IRR 1.12	0.72–1.75	0.602; 0.814
Sexual abuse	Previous MDEs	93	0.0965 (0.2822)	IRR 1.10	0.63–1.91	0.732; 0.814
Emotional neglect	Previous MDEs	93	-0.0006 (0.0872)	IRR 1.00	0.84–1.19	0.994; 0.994
Physical neglect	Previous MDEs	93	-0.3167 (0.1762)	IRR 0.73	0.52–1.03	0.072; 0.241
Emotional abuse	Lifetime failed antidepressant trials	104	0.0304 (0.0721)	IRR 1.03	0.90–1.19	0.673; 0.814
Physical abuse	Lifetime failed antidepressant trials	104	0.1713 (0.0881)	IRR 1.19	1.00–1.41	0.052; 0.208
Sexual abuse	Lifetime failed antidepressant trials	104	0.0728 (0.1408)	IRR 1.08	0.82–1.42	0.605; 0.814
Emotional neglect	Lifetime failed antidepressant trials	104	-0.1752 (0.0596)	IRR 0.84	0.75–0.94	0.003; 0.022
Physical neglect	Lifetime failed antidepressant trials	104	-0.0238 (0.1189)	IRR 0.98	0.77–1.23	0.842; 0.886

All five subscales enter simultaneously, each per 5 points, together with the primary covariates. BH q values use these 20 exploratory conditional coefficients, not the global primary family. The physical-neglect/illness-duration coefficient is conditional on the correlated subscales. Neither physical-abuse association survived this exploratory FDR correction: elapsed ongoing-MDE duration, p=0.023 and q=0.117; failed trials, p=0.052 and q=0.208. These conditional results do not establish an independent physical-abuse association beyond correlated maltreatment dimensions.

Table S10. Full CTQ-by-sex interaction family (exploratory; 28 tests).

Exposure	Outcome	N	Interaction effect	95% CI	p; q
CTQ total	Elapsed ongoing-MDE duration	104	ratio 1.14	0.84–1.56	0.395; 0.915
Trauma load	Elapsed ongoing-MDE duration	104	ratio 1.15	0.88–1.50	0.300; 0.915
Emotional abuse	Elapsed ongoing-MDE duration	104	ratio 1.24	0.86–1.79	0.238; 0.915
Physical abuse	Elapsed ongoing-MDE duration	104	ratio 1.37	0.87–2.15	0.171; 0.915
Sexual abuse	Elapsed ongoing-MDE duration	104	ratio 2.68	0.95–7.56	0.062; 0.915
Emotional neglect	Elapsed ongoing-MDE duration	104	ratio 0.90	0.63–1.29	0.565; 0.915
Physical neglect	Elapsed ongoing-MDE duration	104	ratio 1.19	0.74–1.90	0.472; 0.915
CTQ total	Illness duration	99	-0.20 y	-3.38 to +2.97	0.899; 0.955
Trauma load	Illness duration	99	-0.55 y	-3.73 to +2.63	0.733; 0.955

Exposure	Outcome	N	Interaction effect	95% CI	p; q
Emotional abuse	Illness duration	99	+1.78 y	-2.23 to +5.78	0.380; 0.915
Physical abuse	Illness duration	99	+1.52 y	-4.56 to +7.60	0.621; 0.915
Sexual abuse	Illness duration	99	+3.41 y	-7.28 to +14.10	0.528; 0.915
Emotional neglect	Illness duration	99	-1.93 y	-5.76 to +1.89	0.318; 0.915
Physical neglect	Illness duration	99	-2.23 y	-7.32 to +2.87	0.388; 0.915
CTQ total	Previous MDEs	93	ratio 0.99	0.75–1.30	0.935; 0.955
Trauma load	Previous MDEs	93	ratio 1.01	0.75–1.35	0.955; 0.955
Emotional abuse	Previous MDEs	93	ratio 1.14	0.86–1.52	0.364; 0.915
Physical abuse	Previous MDEs	93	ratio 0.87	0.49–1.52	0.617; 0.915
Sexual abuse	Previous MDEs	93	ratio 0.81	0.11–6.15	0.835; 0.955
Emotional neglect	Previous MDEs	93	ratio 0.93	0.71–1.22	0.604; 0.915
Physical neglect	Previous MDEs	93	ratio 0.97	0.61–1.54	0.887; 0.955
CTQ total	Lifetime failed antidepressant trials	104	ratio 1.08	0.88–1.32	0.479; 0.915
Trauma load	Lifetime failed antidepressant trials	104	ratio 1.02	0.84–1.23	0.846; 0.955
Emotional abuse	Lifetime failed antidepressant trials	104	ratio 1.10	0.90–1.34	0.350; 0.915
Physical abuse	Lifetime failed antidepressant trials	104	ratio 1.09	0.89–1.34	0.417; 0.915
Sexual abuse	Lifetime failed antidepressant trials	104	ratio 0.96	0.56–1.64	0.881; 0.955
Emotional neglect	Lifetime failed antidepressant trials	104	ratio 0.98	0.81–1.19	0.859; 0.955
Physical neglect	Lifetime failed antidepressant trials	104	ratio 1.24	0.89–1.72	0.205; 0.915

Interaction is the female-versus-male difference in exposure slope: years for identity-link illness-duration models, and the ratio of exposure ratios for log-duration/count models. Models include both main effects and the primary covariates. q values use all 28 exploratory interaction tests.

Table S11. Full Minimization/Denial sensitivity analyses (each 28-test family reported separately).

Specification	Exposure	Outcome	N	Effect	95% CI	p; q
M/D-adjusted	CTQ total	Elapsed ongoing-MDE duration	104	+3.6%	-17.4% to +29.9%	0.759; 0.973

Specification	Exposure	Outcome	N	Effect	95% CI	p; q
M/D-adjusted	Trauma load	Elapsed ongoing-MDE duration	104	+1.0%	-14.8% to +19.8%	0.904; 0.973
M/D-adjusted	Emotional abuse	Elapsed ongoing-MDE duration	104	+15.3%	-2.4% to +36.2%	0.094; 0.240
M/D-adjusted	Physical abuse	Elapsed ongoing-MDE duration	104	+55.7%	+29.1% to +87.9%	<0.001; <0.001
M/D-adjusted	Sexual abuse	Elapsed ongoing-MDE duration	104	+67.7%	+32.9% to +111.5%	<0.001; <0.001
M/D-adjusted	Emotional neglect	Elapsed ongoing-MDE duration	104	-25.7%	-40.8% to -6.6%	0.011; 0.053
M/D-adjusted	Physical neglect	Elapsed ongoing-MDE duration	104	-3.9%	-32.5% to +36.9%	0.825; 0.973
M/D-adjusted	CTQ total	Illness duration	99	-0.76 y	-2.59 to +1.06	0.410; 0.765
M/D-adjusted	Trauma load	Illness duration	99	-0.53 y	-2.42 to +1.35	0.577; 0.951
M/D-adjusted	Emotional abuse	Illness duration	99	-0.08 y	-2.01 to +1.86	0.938; 0.973
M/D-adjusted	Physical abuse	Illness duration	99	+0.18 y	-2.99 to +3.36	0.910; 0.973
M/D-adjusted	Sexual abuse	Illness duration	99	+0.27 y	-4.10 to +4.63	0.904; 0.973
M/D-adjusted	Emotional neglect	Illness duration	99	-0.55 y	-2.48 to +1.39	0.576; 0.951
M/D-adjusted	Physical neglect	Illness duration	99	-3.91 y	-7.11 to -0.72	0.017; 0.060
M/D-adjusted	CTQ total	Previous MDEs	93	IRR 0.91	0.79–1.04	0.169; 0.395
M/D-adjusted	Trauma load	Previous MDEs	93	IRR 0.96	0.83–1.12	0.627; 0.973
M/D-adjusted	Emotional abuse	Previous MDEs	93	IRR 1.02	0.88–1.18	0.840; 0.973
M/D-adjusted	Physical abuse	Previous MDEs	93	IRR 1.04	0.75–1.44	0.822; 0.973
M/D-adjusted	Sexual abuse	Previous MDEs	93	IRR 1.00	0.68–1.47	0.985; 0.985
M/D-adjusted	Emotional neglect	Previous MDEs	93	IRR 0.85	0.74–0.98	0.024; 0.068
M/D-adjusted	Physical neglect	Previous MDEs	93	IRR 0.71	0.56–0.90	0.004; 0.023
M/D-adjusted	CTQ total	Lifetime failed antidepressant trials	104	IRR 1.05	0.94–1.17	0.357; 0.734
M/D-adjusted	Trauma load	Lifetime failed antidepressant trials	104	IRR 1.05	0.95–1.16	0.367; 0.734

Specification	Exposure	Outcome	N	Effect	95% CI	p; q
M/D-adjusted	Emotional abuse	Lifetime failed antidepressant trials	104	IRR 1.12	1.01–1.24	0.024; 0.068
M/D-adjusted	Physical abuse	Lifetime failed antidepressant trials	104	IRR 1.26	1.16–1.37	<0.001; <0.001
M/D-adjusted	Sexual abuse	Lifetime failed antidepressant trials	104	IRR 1.25	1.09–1.44	0.002; 0.013
M/D-adjusted	Emotional neglect	Lifetime failed antidepressant trials	104	IRR 0.89	0.81–0.98	0.017; 0.060
M/D-adjusted	Physical neglect	Lifetime failed antidepressant trials	104	IRR 0.99	0.79–1.24	0.928; 0.973
M/D-negative subgroup	CTQ total	Elapsed ongoing-MDE duration	87	+15.5%	+0.4% to +32.9%	0.044; 0.154
M/D-negative subgroup	Trauma load	Elapsed ongoing-MDE duration	87	+8.6%	-6.7% to +26.5%	0.283; 0.566
M/D-negative subgroup	Emotional abuse	Elapsed ongoing-MDE duration	87	+18.9%	-0.7% to +42.5%	0.060; 0.187
M/D-negative subgroup	Physical abuse	Elapsed ongoing-MDE duration	87	+54.5%	+28.5% to +85.8%	<0.001; <0.001
M/D-negative subgroup	Sexual abuse	Elapsed ongoing-MDE duration	87	+62.2%	+28.0% to +105.4%	<0.001; 0.001
M/D-negative subgroup	Emotional neglect	Elapsed ongoing-MDE duration	87	-14.0%	-30.2% to +5.9%	0.154; 0.360
M/D-negative subgroup	Physical neglect	Elapsed ongoing-MDE duration	87	+11.9%	-17.5% to +51.8%	0.465; 0.766
M/D-negative subgroup	CTQ total	Illness duration	82	-0.31 y	-2.28 to +1.65	0.753; 0.992
M/D-negative subgroup	Trauma load	Illness duration	82	+0.02 y	-2.05 to +2.09	0.987; 0.992
M/D-negative subgroup	Emotional abuse	Illness duration	82	+0.01 y	-2.13 to +2.15	0.992; 0.992
M/D-negative subgroup	Physical abuse	Illness duration	82	+0.50 y	-2.77 to +3.78	0.761; 0.992
M/D-negative subgroup	Sexual abuse	Illness duration	82	+1.02 y	-3.01 to +5.06	0.615; 0.957
M/D-negative subgroup	Emotional neglect	Illness duration	82	+0.04 y	-2.38 to +2.46	0.973; 0.992
M/D-negative subgroup	Physical neglect	Illness duration	82	-3.31 y	-7.04 to +0.43	0.082; 0.229
M/D-negative subgroup	CTQ total	Previous MDEs	76	IRR 0.92	0.79–1.08	0.319; 0.595
M/D-negative subgroup	Trauma load	Previous MDEs	76	IRR 0.99	0.85–1.17	0.931; 0.992

Specification	Exposure	Outcome	N	Effect	95% CI	p; q
M/D-negative subgroup	Emotional abuse	Previous MDEs	76	IRR 1.00	0.85–1.18	0.972; 0.992
M/D-negative subgroup	Physical abuse	Previous MDEs	76	IRR 1.05	0.76–1.45	0.789; 0.992
M/D-negative subgroup	Sexual abuse	Previous MDEs	76	IRR 1.02	0.68–1.52	0.942; 0.992
M/D-negative subgroup	Emotional neglect	Previous MDEs	76	IRR 0.87	0.74–1.03	0.111; 0.284
M/D-negative subgroup	Physical neglect	Previous MDEs	76	IRR 0.72	0.55–0.95	0.019; 0.075
M/D-negative subgroup	CTQ total	Lifetime failed antidepressant trials	87	IRR 1.07	0.97–1.17	0.196; 0.422
M/D-negative subgroup	Trauma load	Lifetime failed antidepressant trials	87	IRR 1.04	0.95–1.15	0.366; 0.641
M/D-negative subgroup	Emotional abuse	Lifetime failed antidepressant trials	87	IRR 1.15	1.04–1.26	0.005; 0.029
M/D-negative subgroup	Physical abuse	Lifetime failed antidepressant trials	87	IRR 1.26	1.17–1.36	<0.001; <0.001
M/D-negative subgroup	Sexual abuse	Lifetime failed antidepressant trials	87	IRR 1.24	1.08–1.42	0.003; 0.020
M/D-negative subgroup	Emotional neglect	Lifetime failed antidepressant trials	87	IRR 0.87	0.78–0.96	0.008; 0.037
M/D-negative subgroup	Physical neglect	Lifetime failed antidepressant trials	87	IRR 0.95	0.75–1.21	0.676; 0.992

M/D-adjusted models add the 0–3 M/D score; M/D-negative models retain only score=0. Each specification has its own exploratory 28-test BH family. Exposure units and base covariates match the primary analysis.

Table S12. Restricted-cubic-spline linearity assessment (exploratory family of 28 tests).

Exposure	Outcome	N	Knots	Nonlinear term/note	p	q
CTQ total	Elapsed ongoing-MDE duration	104	28.30, 49.00, 64.00	nonlinear β=0.0469 (SE 0.0156)	0.003	0.031
Trauma load	Elapsed ongoing-MDE duration	104	0.00, 2.00, 3.00	nonlinear β=0.5550 (SE 0.1398)	<0.001	0.004
Emotional abuse	Elapsed ongoing-MDE duration	104	5.00, 8.00, 17.00	nonlinear β=0.0087 (SE 0.1245)	0.944	1.000
Physical abuse	Elapsed ongoing-MDE duration	104	5.00, 5.00, 10.70	insufficient distinct knots	—	1.000
Sexual abuse	Elapsed ongoing-MDE duration	104	5.00, 5.00, 9.00	insufficient distinct knots	—	1.000

Exposure	Outcome	N	Knots	Nonlinear term/note	p	q
Emotional neglect	Elapsed ongoing-MDE duration	104	6.00, 15.00, 21.70	nonlinear β=-0.0551 (SE 0.0561)	0.328	0.919
Physical neglect	Elapsed ongoing-MDE duration	104	5.00, 9.00, 16.00	nonlinear β=0.2320 (SE 0.0897)	0.011	0.063
CTQ total	Illness duration	99	27.80, 48.00, 61.00	nonlinear β=0.0446 (SE 0.1915)	0.816	1.000
Trauma load	Illness duration	99	0.00, 2.00, 3.00	nonlinear β=1.2090 (SE 1.8638)	0.518	1.000
Emotional abuse	Illness duration	99	5.00, 8.00, 17.00	nonlinear β=-0.9130 (SE 1.5200)	0.550	1.000
Physical abuse	Illness duration	99	5.00, 5.00, 11.20	insufficient distinct knots	—	1.000
Sexual abuse	Illness duration	99	5.00, 5.00, 9.00	insufficient distinct knots	—	1.000
Emotional neglect	Illness duration	99	6.00, 15.00, 22.00	nonlinear β=-0.6655 (SE 0.5811)	0.255	0.793
Physical neglect	Illness duration	99	5.00, 9.00, 15.00	nonlinear β=0.3067 (SE 1.1392)	0.788	1.000
CTQ total	Previous MDEs	93	27.20, 47.00, 58.00	nonlinear β=0.0008 (SE 0.0101)	0.934	1.000
Trauma load	Previous MDEs	93	0.00, 2.00, 3.00	nonlinear β=0.1143 (SE 0.1314)	0.384	0.979
Emotional abuse	Previous MDEs	93	5.00, 7.00, 17.00	nonlinear β=-0.0339 (SE 0.1671)	0.839	1.000
Physical abuse	Previous MDEs	93	5.00, 5.00, 9.80	insufficient distinct knots	—	1.000
Sexual abuse	Previous MDEs	93	5.00, 5.00, 8.80	insufficient distinct knots	—	1.000
Emotional neglect	Previous MDEs	93	6.00, 15.00, 21.80	nonlinear β=-0.1078 (SE 0.0450)	0.017	0.078
Physical neglect	Previous MDEs	93	5.00, 9.00, 13.00	nonlinear β=-0.0301 (SE 0.0762)	0.693	1.000
CTQ total	Lifetime failed antidepressant trials	104	28.30, 49.00, 64.00	nonlinear β=0.0228 (SE 0.0112)	0.042	0.157
Trauma load	Lifetime failed antidepressant trials	104	0.00, 2.00, 3.00	nonlinear β=0.2117 (SE 0.0830)	0.011	0.063
Emotional abuse	Lifetime failed antidepressant trials	104	5.00, 8.00, 17.00	nonlinear β=0.1383 (SE 0.0689)	0.045	0.157
Physical abuse	Lifetime failed antidepressant trials	104	5.00, 5.00, 10.70	insufficient distinct knots	—	1.000
Sexual abuse	Lifetime failed antidepressant trials	104	5.00, 5.00, 9.00	insufficient distinct knots	—	1.000
Emotional neglect	Lifetime failed antidepressant trials	104	6.00, 15.00, 21.70	nonlinear β=0.0193 (SE 0.0282)	0.493	1.000
Physical neglect	Lifetime failed antidepressant trials	104	5.00, 9.00, 16.00	nonlinear β=0.1568 (SE 0.0519)	0.003	0.031

The three-knot restricted cubic spline adds one nonlinear basis term to the linear exposure effect. Wald p values test that nonlinear coefficient. BH correction retains the 28-test exploratory family, assigning p=1 to nonestimable knot configurations; those table cells show a dash rather than an estimate. Sparse/floor exposure and modest sample size limit interpretation.

Table S13. Influence and bootstrap analyses for the principal dimension-specific findings.

Exposure	Outcome	Method	Estimate/range	p-value range	Iterations p≥0.05	Influence metric/interval
Physical abuse	Elapsed ongoing-MDE duration	Leave-one-out	47.400 to 61.584	<0.001 to 0.002	0	max absolute change=11.26
Sexual abuse	Elapsed ongoing-MDE duration	Leave-one-out	53.602 to 66.577	<0.001 to 0.060	1	max absolute change=6.79
Emotional neglect	Elapsed ongoing-MDE duration	Leave-one-out	-27.575 to -21.813	<0.001 to 0.007	0	max absolute change=3.29
Physical abuse	Lifetime failed antidepressant trials	Leave-one-out	1.178 to 1.220	<0.001 to 0.004	0	max absolute change=0.02
Sexual abuse	Lifetime failed antidepressant trials	Leave-one-out	1.068 to 1.215	<0.001 to 0.756	102	max absolute change=0.10
Emotional neglect	Lifetime failed antidepressant trials	Leave-one-out	0.833 to 0.872	<0.001 to 0.004	0	max absolute change=0.02
Physical abuse	Elapsed ongoing-MDE duration	2,000 bootstrap resamples	median 50.716	—	—	95% percentile CI 22.422 to 80.941
Sexual abuse	Elapsed ongoing-MDE duration	2,000 bootstrap resamples	median 59.515	—	—	95% percentile CI 6.214 to 126.041
Emotional neglect	Elapsed ongoing-MDE duration	2,000 bootstrap resamples	median -25.205	—	—	95% percentile CI -36.930 to -10.939
Physical abuse	Lifetime failed antidepressant trials	2,000 bootstrap resamples	median 1.195	—	—	95% percentile CI 1.079 to 1.313
Sexual abuse	Lifetime failed antidepressant trials	2,000 bootstrap resamples	median 1.166	—	—	95% percentile CI 0.825 to 1.441
Emotional neglect	Lifetime failed antidepressant trials	2,000 bootstrap resamples	median 0.852	—	—	95% percentile CI 0.774 to 0.943

Duration effects and changes are percentage points on the conditional geometric-mean scale; failed-trial effects are IRRs. Leave-one-out omits each of 104 participants in turn; influence is the maximum absolute difference from the full-sample estimate. Bootstrap intervals are percentile intervals from 2,000 valid resamples. These robustness summaries have no separate FDR family.

Table S14. Negative-binomial sensitivities for both count outcomes (exploratory family of 14 tests).

Exposure	Outcome	N	IRR	95% CI	p; q	Dispersion α
CTQ total	Previous MDEs	93	0.96	0.85–1.08	0.494; 0.857	0.205
Trauma load	Previous MDEs	93	1.00	0.88–1.14	0.965; 0.965	0.208
Emotional abuse	Previous MDEs	93	1.04	0.89–1.21	0.612; 0.857	0.206

Exposure	Outcome	N	IRR	95% CI	p; q	Dispersion α
Physical abuse	Previous MDEs	93	1.06	0.86–1.32	0.591; 0.857	0.206
Sexual abuse	Previous MDEs	93	1.05	0.73–1.50	0.806; 0.894	0.208
Emotional neglect	Previous MDEs	93	0.91	0.79–1.05	0.191; 0.491	0.198
Physical neglect	Previous MDEs	93	0.78	0.61–0.98	0.035; 0.164	0.182
CTQ total	Lifetime failed antidepressant trials	104	0.99	0.92–1.07	0.830; 0.894	0.032
Trauma load	Lifetime failed antidepressant trials	104	0.99	0.91–1.08	0.790; 0.894	0.032
Emotional abuse	Lifetime failed antidepressant trials	104	1.07	0.96–1.18	0.210; 0.491	0.028
Physical abuse	Lifetime failed antidepressant trials	104	1.20	1.06–1.35	0.004; 0.028	0.015
Sexual abuse	Lifetime failed antidepressant trials	104	1.16	0.95–1.43	0.147; 0.491	0.027
Emotional neglect	Lifetime failed antidepressant trials	104	0.85	0.78–0.93	<0.001; 0.006	0.005
Physical neglect	Lifetime failed antidepressant trials	104	0.92	0.79–1.07	0.281; 0.562	0.029

NB2 maximum-likelihood models use the primary covariates; extraction block is absent where inestimable. q values are BH-adjusted within these 14 tests. All models converged; near-zero α approaches the Poisson model.

Table S15. Count outcomes additionally adjusted for illness duration (exploratory family of 14 tests).

Exposure	Outcome	N	IRR	95% CI	p; q
CTQ total	Previous MDEs	93	0.97	0.88–1.06	0.501; 0.878
Trauma load	Previous MDEs	93	1.00	0.90–1.10	0.948; 0.948
Emotional abuse	Previous MDEs	93	1.04	0.91–1.18	0.584; 0.909
Physical abuse	Previous MDEs	93	1.03	0.85–1.25	0.754; 0.948
Sexual abuse	Previous MDEs	93	1.03	0.62–1.70	0.919; 0.948
Emotional neglect	Previous MDEs	93	0.90	0.79–1.01	0.080; 0.371
Physical neglect	Previous MDEs	93	0.90	0.74–1.10	0.311; 0.727
CTQ total	Lifetime failed antidepressant trials	99	0.99	0.86–1.14	0.879; 0.948
Trauma load	Lifetime failed antidepressant trials	99	0.98	0.88–1.10	0.772; 0.948

Exposure	Outcome	N	IRR	95% CI	p; q
Emotional abuse	Lifetime failed antidepressant trials	99	1.06	0.96–1.18	0.252; 0.706
Physical abuse	Lifetime failed antidepressant trials	99	1.19	1.09–1.29	<0.001; <0.001
Sexual abuse	Lifetime failed antidepressant trials	99	1.16	0.92–1.48	0.206; 0.706
Emotional neglect	Lifetime failed antidepressant trials	99	0.85	0.77–0.95	0.002; 0.017
Physical neglect	Lifetime failed antidepressant trials	99	0.91	0.71–1.17	0.458; 0.878

Poisson models add years since first MDE to the primary covariates. q values use these 14 exploratory tests; adjustment may control exposure opportunity but may also condition on a mediator.

Table S16. All models restricted to participants with at least two recorded failed antidepressant trials.

Exposure	Outcome	N	Effect	95% CI	p; q
CTQ total	Elapsed ongoing-MDE duration	103	+1.8%	-16.3% to +23.9%	0.855; 0.957
Trauma load	Elapsed ongoing-MDE duration	103	-0.3%	-14.0% to +15.6%	0.968; 0.968
Emotional abuse	Elapsed ongoing-MDE duration	103	+13.0%	-4.1% to +33.3%	0.143; 0.499
Physical abuse	Elapsed ongoing-MDE duration	103	+51.6%	+25.0% to +83.8%	<0.001; <0.001
Sexual abuse	Elapsed ongoing-MDE duration	103	+62.5%	+28.7% to +105.3%	<0.001; <0.001
Emotional neglect	Elapsed ongoing-MDE duration	103	-24.4%	-37.1% to -9.1%	0.003; 0.018
Physical neglect	Elapsed ongoing-MDE duration	103	-4.6%	-30.5% to +31.0%	0.768; 0.957
CTQ total	Illness duration	98	+0.06 y	-1.44 to +1.56	0.938; 0.968
Trauma load	Illness duration	98	+0.23 y	-1.55 to +2.01	0.799; 0.957
Emotional abuse	Illness duration	98	+0.47 y	-1.41 to +2.35	0.624; 0.957
Physical abuse	Illness duration	98	+0.71 y	-2.44 to +3.87	0.654; 0.957
Sexual abuse	Illness duration	98	+1.19 y	-2.28 to +4.66	0.498; 0.957
Emotional neglect	Illness duration	98	+0.33 y	-1.65 to +2.32	0.739; 0.957
Physical neglect	Illness duration	98	-2.60 y	-5.74 to +0.54	0.104; 0.416
CTQ total	Previous MDEs	92	IRR 0.97	0.86–1.09	0.587; 0.957
Trauma load	Previous MDEs	92	IRR 1.01	0.87–1.17	0.889; 0.958

Exposure	Outcome	N	Effect	95% CI	p; q
Emotional abuse	Previous MDEs	92	IRR 1.05	0.91–1.21	0.487; 0.957
Physical abuse	Previous MDEs	92	IRR 1.07	0.78–1.48	0.665; 0.957
Sexual abuse	Previous MDEs	92	IRR 1.05	0.68–1.61	0.824; 0.957
Emotional neglect	Previous MDEs	92	IRR 0.92	0.81–1.05	0.201; 0.585
Physical neglect	Previous MDEs	92	IRR 0.79	0.62–0.99	0.041; 0.192
CTQ total	Lifetime failed antidepressant trials	103	IRR 0.98	0.85–1.13	0.798; 0.957
Trauma load	Lifetime failed antidepressant trials	103	IRR 0.98	0.88–1.09	0.691; 0.957
Emotional abuse	Lifetime failed antidepressant trials	103	IRR 1.06	0.96–1.18	0.271; 0.689
Physical abuse	Lifetime failed antidepressant trials	103	IRR 1.19	1.10–1.30	<0.001; <0.001
Sexual abuse	Lifetime failed antidepressant trials	103	IRR 1.15	0.92–1.44	0.209; 0.585
Emotional neglect	Lifetime failed antidepressant trials	103	IRR 0.84	0.76–0.93	<0.001; 0.003
Physical neglect	Lifetime failed antidepressant trials	103	IRR 0.90	0.71–1.15	0.397; 0.926

The single participant with a recorded count of 1 is excluded. This is an exploratory family of 28 tests with within-family BH q values.

Table S17. Failed-trial count additionally adjusted for log ongoing-MDE duration.

Exposure	N	IRR	95% CI	p; q
CTQ total	104	0.99	0.88–1.11	0.852; 0.852
Trauma load	104	0.99	0.90–1.09	0.820; 0.852
Emotional abuse	104	1.05	0.94–1.17	0.372; 0.694
Physical abuse	104	1.15	1.03–1.28	0.013; 0.055
Sexual abuse	104	1.09	0.87–1.36	0.438; 0.694
Emotional neglect	104	0.87	0.78–0.97	0.016; 0.055
Physical neglect	104	0.93	0.75–1.15	0.495; 0.694

Models add log elapsed current-episode duration to the primary covariates. This adjustment tests overlap between the two cross-sectional outcomes and is not a causal direct-effect analysis. q values use seven exploratory tests. The physical-abuse estimate attenuated to IRR=1.15 (95% CI, 1.03–1.28; p=0.013; q=0.055) and did not survive this exploratory FDR correction. This analysis therefore does not establish an association independent of elapsed ongoing-episode duration.

Table S18. All models additionally adjusted for an aggregate psychiatric-comorbidity indicator.

Exposure	Outcome	N	Effect	95% CI	p; q
CTQ total	Elapsed ongoing-MDE duration	103	+0.4%	-18.0% to +23.0%	0.967; 0.989
Trauma load	Elapsed ongoing-MDE duration	103	-1.6%	-15.3% to +14.4%	0.835; 0.989
Emotional abuse	Elapsed ongoing-MDE duration	103	+11.9%	-5.2% to +32.2%	0.181; 0.564
Physical abuse	Elapsed ongoing-MDE duration	103	+52.0%	+27.2% to +81.6%	<0.001; <0.001
Sexual abuse	Elapsed ongoing-MDE duration	103	+60.7%	+27.9% to +101.8%	<0.001; 0.001
Emotional neglect	Elapsed ongoing-MDE duration	103	-25.2%	-37.8% to -10.0%	0.002; 0.014
Physical neglect	Elapsed ongoing-MDE duration	103	-7.0%	-32.9% to +28.9%	0.660; 0.989
CTQ total	Illness duration	98	-0.21 y	-1.75 to +1.33	0.786; 0.989
Trauma load	Illness duration	98	+0.03 y	-1.76 to +1.82	0.975; 0.989
Emotional abuse	Illness duration	98	+0.29 y	-1.57 to +2.15	0.756; 0.989
Physical abuse	Illness duration	98	+0.69 y	-2.48 to +3.87	0.665; 0.989
Sexual abuse	Illness duration	98	+1.29 y	-1.56 to +4.13	0.371; 0.866
Emotional neglect	Illness duration	98	+0.02 y	-2.01 to +2.06	0.982; 0.989
Physical neglect	Illness duration	98	-3.50 y	-6.64 to -0.35	0.030; 0.119
CTQ total	Previous MDEs	92	IRR 0.96	0.85–1.08	0.461; 0.989
Trauma load	Previous MDEs	92	IRR 1.00	0.87–1.15	0.989; 0.989
Emotional abuse	Previous MDEs	92	IRR 1.04	0.91–1.19	0.569; 0.989
Physical abuse	Previous MDEs	92	IRR 1.07	0.76–1.51	0.696; 0.989
Sexual abuse	Previous MDEs	92	IRR 1.05	0.64–1.73	0.837; 0.989
Emotional neglect	Previous MDEs	92	IRR 0.91	0.80–1.04	0.154; 0.538
Physical neglect	Previous MDEs	92	IRR 0.75	0.60–0.95	0.018; 0.086
CTQ total	Lifetime failed antidepressant trials	103	IRR 0.99	0.86–1.14	0.879; 0.989
Trauma load	Lifetime failed antidepressant trials	103	IRR 0.99	0.89–1.10	0.802; 0.989

Exposure	Outcome	N	Effect	95% CI	p; q
Emotional abuse	Lifetime failed antidepressant trials	103	IRR 1.06	0.95–1.18	0.292; 0.744
Physical abuse	Lifetime failed antidepressant trials	103	IRR 1.18	1.08–1.29	<0.001; 0.002
Sexual abuse	Lifetime failed antidepressant trials	103	IRR 1.17	0.91–1.50	0.226; 0.634
Emotional neglect	Lifetime failed antidepressant trials	103	IRR 0.85	0.77–0.94	0.002; 0.013
Physical neglect	Lifetime failed antidepressant trials	103	IRR 0.93	0.73–1.18	0.530; 0.989

A binary indicator of any recorded psychiatric comorbidity is added to the primary covariates; one participant lacks this indicator. Diagnostic subtypes were not reliably harmonized. q values use this exploratory 28-test family. The indicator uses 0 versus nonzero numeric source codes, retaining missing values. It represents recorded aggregate comorbidity rather than independently validated disorder-specific diagnoses.
